# Impact of Beneficial Microorganisms on Strawberry Growth, Fruit Production, Nutritional Quality, and Volatilome

**DOI:** 10.3389/fpls.2018.01611

**Published:** 2018-11-16

**Authors:** Valeria Todeschini, Nassima AitLahmidi, Eleonora Mazzucco, Francesco Marsano, Fabio Gosetti, Elisa Robotti, Elisa Bona, Nadia Massa, Laurent Bonneau, Emilio Marengo, Daniel Wipf, Graziella Berta, Guido Lingua

**Affiliations:** ^1^Dipartimento di Scienze ed Innovazione Tecnologica, Università del Piemonte Orientale, Vercelli, Italy; ^2^Agroécologie, AgroSup Dijon, CNRS, INRA, Univ. Bourgogne Franche-Comté, Dijon, France; ^3^Dipartimento di Scienze ed Innovazione Tecnologica, Università del Piemonte Orientale, Alessandria, Italy

**Keywords:** PGPB, AMF, strawberry, nutritional quality, volatile compounds, sustainable agriculture, biofertilizers, chemometrics

## Abstract

Arbuscular mycorrhizal fungi (AMF) colonize the roots of most terrestrial plant species, improving plant growth, nutrient uptake and biotic/abiotic stress resistance and tolerance. Similarly, plant growth promoting bacteria (PGPB) enhance plant fitness and production. In this study, three different AMF (*Funneliformis mosseae, Septoglomus viscosum*, and *Rhizophagus irregularis*) were used in combination with three different strains of *Pseudomonas* sp. (19Fv1t, 5Vm1K and Pf4) to inoculate plantlets of *Fragaria* × *ananassa* var. Eliana F1. The effects of the different fungus/bacterium combinations were assessed on plant growth parameters, fruit production and quality, including health-promoting compounds. Inoculated and uninoculated plants were maintained in a greenhouse for 4 months and irrigated with a nutrient solution at two different phosphate levels. The number of flowers and fruits were recorded weekly. At harvest, fresh and dry weights of roots and shoots, mycorrhizal colonization and concentration of leaf photosynthetic pigments were measured in each plant. The following fruit parameters were recorded: pH, titratable acids, concentration of organic acids, soluble sugars, ascorbic acids, and anthocyanidins; volatile and elemental composition were also evaluated. Data were statistically analyzed by ANOVA and PCA/PCA-DA. Mycorrhizal colonization was higher in plants inoculated with *R. irregularis*, followed by *F. mosseae* and *S. viscosum*. In general, AMF mostly affected the parameters associated with the vegetative portion of the plant, while PGPB were especially relevant for fruit yield and quality. The plant physiological status was differentially affected by inoculations, resulting in enhanced root and shoot biomass. Inoculation with Pf4 bacterial strain increased flower and fruit production per plant and malic acid content in fruits, while decreased the pH value, regardless of the used fungus. Inoculations affected fruit nutritional quality, increasing sugar and anthocyanin concentrations, and modulated pH, malic acid, volatile compounds and elements. In the present study, we show for the first time that strawberry fruit concentration of some elements and/or volatiles can be affected by the presence of specific beneficial soil microorganisms. In addition, our results indicated that it is possible to select the best plant-microorganism combination for field applications, and improving fruit production and quality, also in terms of health promoting properties.

## Introduction

Plants interact with a huge variety of beneficial microorganisms such as arbuscular mycorrhizal fungi (AMF) and plant growth-promoting bacteria (PGPB), which can improve both plant fitness and production.

AMF belong to the Glomeromycotina subphylum (Spatafora et al., 2016) and are symbiotically associated to the roots of the majority of land plants, including the main crop species. They play an ecologically important role and provide various ecosystem services such as the improvement of nutrient uptake, soil aggregation, and protection against biotic and abiotic stress (Lingua et al., 2008; Gianinazzi et al., 2010; Smith et al., 2010; Antunes et al., 2012; Boyer et al., 2015).

PGPB comprise different functional and taxonomic groups (Ghosh et al., 2003), among which *Pseudomonas fluorescens* is one of the most extensively studied species (Duijff et al., 1997; Vazquez et al., 2000). They directly enhance plant growth by a variety of mechanisms, such as mobilization of soil nutrients, atmospheric nitrogen fixation, phosphorus solubilization, and phytohormone synthesis, especially IAA (Indole-3-Acetic Acid) (Glick, 1995). PGPB can also act indirectly, suppressing phytopathogens (Benizri et al., 2001). Some fluorescent pseudomonads were shown to improve mycorrhizal root colonization (Gamalero et al., 2008, 2010), extraradical hyphal growth (Mugnier and Mosse, 1987) and AMF spore germination (Frey-Klett et al., 2007), functioning as mycorrhiza helper bacteria (MHB). Conversely, AMF can influence the chemical composition of root exudates, which are a major nutrient source for the bacteria in the rhizosphere (Hegde et al., 1999; Artursson et al., 2006).

The strawberry, *Fragaria* × *ananassa* Duch., belonging to the family Rosaceae, is one of the most cultivated berry crops in Europe and all around the world (Akhatou and Fernandez-Recamales, 2014a; Prat et al., 2014). The large size and the deep red color of its false fruit, besides its characteristic aroma (Ulrich et al., 2007), are the results of the crossing between the North American strawberry *Fragaria virginiana* Mill. with the Chilean strawberry *Fragaria chiloensis* (L.) Mill. (Darrow and Wallace, 1966). The consumption of this fruit, fresh, frozen, or processed (juices, jams, yogurts, etc.), in the daily diet is an important source of healthy compounds (Tulipani et al., 2009) such as fibers, vitamins (in particular C and B9), minerals (mainly potassium and magnesium), and antioxidants (flavonoids, phenolic acids, and ellagitannins) that can prevent or reduce certain types of cancer, cardiovascular diseases, obesity, type II diabetes and cellular damage induced by reactive oxygen species (ROS) (Olsson et al., 2004; Wang and Lewers, 2007; He and Giusti, 2010; Giampieri et al., 2012). Therefore, the strawberry nutritional values and beneficial properties of its bioactive components (a heterogeneous group of biologically active non-nutrients, mainly represented by polyphenols), together with a complex blend of volatile organic compounds, make it a highly appreciated fruit (Diamanti et al., 2012). Among polyphenols, flavonoids, especially anthocyanins in the form of pelargonidin and cyanidin derivatives, are responsible for the strawberry bright red color. These two characteristics are markers of ripening, a process in which anthocyanin accumulation in the fruit flesh and/or skin is highest (da Silva Pinto et al., 2008; Giampieri et al., 2012; Jaakola, 2013). Moreover, the key role of these molecules, both in fruit tolerance to the environmental stresses and in ameliorating post-harvest quality and shelf life, is well-known (Xu et al., 2014). The biosynthesis and accumulation of anthocyanins in fruits are regulated by different factors: genetic (e.g., MYB genes), environmental (light exposure, temperature, nutrient availability such as nitrogen and calcium) and developmental (sugars, in particular sucrose, and hormones including abscisic acid, jasmonate, and ethylene) (Carbone et al., 2009; Jaakola, 2013).

Texture (firmness, juiciness, and crispness), color, sweetness and aroma are the most important quality indicators for strawberry consumers (Christensen, 1983; Colquhon et al., 2012; Negri et al., 2015). Sugars and organic acids are the principal soluble component in ripe fruit (Perez et al., 1992; Moing et al., 2001). The ratio between sugars and acids strongly affects fruit aroma and flavor (Montero et al., 1996; Kallio et al., 2000). For this reason it is considered an index of fruit quality and its variations depend on plant genotype, ripening stage, climatic factors, and soil type (Kallio et al., 2000; Akhatou and Fernandez-Recamales, 2014b; Valentinuzzi et al., 2014). Fructose, glucose, and sucrose, the most abundant soluble solids in strawberries, are responsible for fruit sweetness (Perez et al., 1997). Sugars are involved in many biological processes: in particular, sucrose is involved in fruit ripeness (Jia et al., 2011) and anthocyanin synthesis (Gollop et al., 2002), while fructose is a precursor of the volatile compounds furanones (Sanz et al., 1997; Forney et al., 2000), that contribute to the caramel aroma of the fruit (Prat et al., 2014). Organic acids are important for the maintenance of nutritional values and fruit quality (Mikulic-Petkovsek et al., 2012), besides their involvement in the gelling process of pectin (Cordenunsi et al., 2002). The most abundant organic acid in strawberry is citric acid, whose concentration is responsible for about 92% of total acidity (Cordenunsi et al., 2002; Mahmood et al., 2012), while malic, tartaric, shikimic, quinic, and fumaric acids are present in very small amounts.

Strawberry aroma is mainly due to the complex blend of volatile organic compounds (known as volatilome—Bicchi and Maffei, 2012; Ulrich et al., 2018), which also contribute to the fruit flavor (Forney et al., 2000; Forney, 2001; Prat et al., 2014; Schwieterman et al., 2014). Although these molecules have a big impact on fruit sensorial qualities, they are found in very low concentrations (ranging between about 0.001 and 0.01% of fruit fresh weight) (Buttery, 1981). Approximately 360 volatile molecules seem to be involved in odor and aroma perception of *F*. × *ananassa* fruits by humans, with esters, aldehydes, ketones, alcohols, furanones, and terpenoids being the most representative (Larsen et al., 1992; Prat et al., 2014). The volatile profile changes according to cultivars, stage of ripeness and environmental conditions (Forney et al., 2000). Also soil microorganisms, such as AMF and PGPB (that are considered either as “biofertilizers”—Vessey, 2003; Rai, 2006—or “bioprotectors”—Borowicz, 2001; Antunes et al., 2012) can modulate the volatile profile in various plant species (Bona et al., 2016).

While the effects of bacterial and fungal inocula on the physiology and biochemistry of the whole plant have been investigated in several studies (reviewed in Vessey, 2003; Saharan and Nehra, 2011; Zeng et al., 2013; Sbrana et al., 2014 and references therein), only in recent years the attention has been focused both on sensorial qualities and nutritional/nutraceutical values (mainly healthy compounds) of the edible portions of various crop plants (Nautiyal et al., 2008; Giovannetti et al., 2013; Berta et al., 2014; Bona et al., 2015; Hart et al., 2015). Although several studies (about 70–80) describing strawberry aroma, flavor, sweetness and production of health-promoting compounds in uninoculated plants have been published before (Olsson et al., 2004; Tulipani et al., 2008; Akhatou and Fernandez-Recamales, 2014a,b; Prat et al., 2014; Valentinuzzi et al., 2014; Negri et al., 2015), those related to inoculated ones are fewer (Castellanos-Morales et al., 2010; Lingua et al., 2013; Palencia et al., 2013; Pešaković et al., 2013; Bona et al., 2015; Boyer et al., 2015) and no one concerned the volatile profile. In strawberry, it has been shown that AM colonization stimulates plant growth (Hršelová et al., 1990), enhances photosynthesis (Borkowska, 2002), induces early flowering and fruit production (Lu and Koide, 1994; Gaur et al., 2000; Poulton et al., 2002; Sohn et al., 2003), increases the concentration of sugar and anthocyanins (Castellanos-Morales et al., 2010), thereby resulting in fruits of higher quality. On the contrary, only few studies have been performed to investigate the effect of PGPB on strawberry (Lingua et al., 2013; Bona et al., 2015). A synergistic effect on strawberry growth following co-inoculation with AMF and *Pseudomonas putida* and a stimulation of AMF root colonization by *Agrobacterium radiobacter* have previously been reported (Vosatka et al., 1992, 2000). Therefore, in this work we wanted to test the effects of three AMF and three PGPB strains on *F*. × *ananassa* (var. Eliana F1) plants grown in a greenhouse at low P level. Both plant (mycorrhizal colonization, root and shoot dry weight, number of flowers, chlorophyll concentration, fruit number, weight, and size) and fruit (pH, total acidity; organic acid, sugar, and anthocyanidin concentration; volatile profile and element composition) parameters were considered in order to establish which fungus/bacterium combination gave the best results especially in terms of fruit quality and production. Moreover, by using a multivariate statistical approach (Principal Component Analysis-Discriminant Analysis, PCA-DA) it was possible to determine which bacterium or fungus induced changes on each parameter. To our knowledge the present study shows, for the first time, how different soil beneficial microorganisms (AMF and PGPB) can affect the volatile profile and chemical element composition in strawberry fruits.

## Materials and methods

### Plant material and growth conditions

Seedlings of *Fragaria* × *ananassa* Duch var. Elyana F1 with continuous flowering habit, were transplanted in plastic pots (400 mL) in a soil (Brill Ortopack, Agrochimica, Bolzano, Italy; pH 5.5–6.5) previously sterilized by flowing steam (101°C for 1 h), and kept in a greenhouse for rooting. After 1 month, they were transplanted in new plastic pots (900 mL) in a 2/1 v/v mixture of sterile soil (the same used in the first transplant) and sand (sterilized in oven at 180°C for 3 h), and inoculated or not with one of three different AMF in combination with one of three different bacterial strains (Table 1). Plants were initially irrigated 3 times per week. When the plants began to produce fruits, they were transplanted again in plastic pots of 3 L capacity. Starting from 1 week after the last transplant, they were irrigated daily: once per week with a Long Ashton (LA) nutrient solution (Trotta et al., 1996), and with tap water on the other days. In particular, half of the control plants (C) were watered with LA 32 μM phosphate, while the remaining controls (C-P) and all the inoculated plants were fed with LA 16 μM phosphate (Table 1) until harvest. Strawberry plants were maintained in greenhouse for 16 weeks.

**Table 1:** Summary of the different plant treatments included in the experimental design.

**Fungal isolate**	**Bacterial strain**	**P concentration in LA solution**	**Treatment abbreviation**
No fungus	No bacteria	32 μM	C
No fungus	No bacteria	16 μM	C-P
*Funneliformis mosseae* BEG12 (Fm)	*Pseudomonas fluorescens* 19Fv1t (19Fv)	16 μM	Fm19Fv
*Funneliformis mosseae* BEG12 (Fm)	*Pseudomonas* sp. 5Vm1K (5Vm)	16 μM	Fm5Vm
*Funneliformis mosseae* BEG12 (Fm)	*Pseudomonas fluorescens* Pf4 (Pf4)	16 μM	FmPf4
*Septoglomus viscosum* (Sv)	*Pseudomonas fluorescens* 19Fv1t (19Fv)	16 μM	Sv19Fv
*Septoglomus viscosum* (Sv)	*Pseudomonas* sp. 5Vm1K (5Vm)	16 μM	Sv5Vm
*Septoglomus viscosum* (Sv)	*Pseudomonas fluorescens* Pf4 (Pf4)	16 μM	SvPf4
*Rhizophagus irregularis* DAOM197-198 (Ri)	*Pseudomonas fluorescens* 19Fv1t (19Fv)	16 μM	Ri19Fv
*Rhizophagus irregularis* DAOM197-198 (Ri)	*Pseudomonas* sp. 5Vm1K (5Vm)	16 μM	Ri5Vm
*Rhizophagus irregularis* DAOM197-198 (Ri)	*Pseudomonas fluorescens* Pf4 (Pf4)	16 μM	RiPf4

*Eleven treatments, each with 10 biological repetitions, for a total of 110 plants. C, uninoculated plants grown at 32 μM P; C-P, uninoculated plants grown at 16 μM P; Fm19Fv, Fm5Vm, FmPf4, plants grown at 16 μM P and inoculated with F. mosseae in combination with the three different PGPB strains 19Fv1t, 5Vm1k and Pf4, respectively; Sv19Fv, Sv5Vm, SvPf4, plants grown at 16 μM P and inoculated with S. viscosum in combination with the three different PGPB strains 19Fv1t, 5Vm1k, and Pf4, respectively; Ri19Fv, Ri5Vm, RiPf4, plants grown at 16 μM P and inoculated with R. irregularis in combination with the three different PGPB strains 19Fv1t, 5Vm1k, and Pf4, respectively*.

### Fungal inocula and mycorrhizal colonization

The arbuscular mycorrhizal fungus *Rhizophagus irregularis* (Ri) (DAOM197-198) was provided by INRA (Recorbet and Bernaud, Dijon). *Funneliformis mosseae* (Fm) (BEG12) was provided by the European Bank of Glomales (Dijon). *Septoglomus viscosum* (Sv), collected from an Italian soil (Tuscany, Italy), was produced and provided by Mybasol S.r.l. (Alessandria, Italy). The three inocula, prepared as a mixture of soil, mycorrhizal roots, hyphae, and spores, were mixed (11% v/v) with the plant growth medium.

At harvest, mycorrhizal colonization was assessed on 60 root pieces (1 cm long) randomly chosen from each plant. Samples were cleared in 10% KOH for 30 min at 60°C, stained with 1% methyl blue in lactic acid and mounted on a slide. Frequency of mycorrhization (F%), percentage of colonized root tissue (M%), and abundance of arbuscules (A%) and vesicles (V%) were calculated according to Trouvelot et al. (1986).

### Bacterial inocula

*Pseudomonas fluorescens* strain Pf4 (Pf4) was isolated from a forest soil located in Sassello (Savona, Italy) and characterized by Berta et al. (2014). *Pseudomonas* sp. 5Vm1K (5Vm) was isolated from the rhizosphere of blueberry plants grown in a larch woodland (Bellino, Cuneo, Italy) and characterized as described by Bona et al. (2015). *P. fluorescens* strain 19Fv1t (19Fv) was provided by Mybasol s.r.l (Alessandria, Italy) and characterized by Bona et al. (2017). Bacterial 16S rDNA sequences were deposited in the NCBI database GenBank with the accession numbers KF234076, KF233995, KF752592 for Pf4, 5Vm, and 19Fv, respectively.

Bacterial cells were grown on tryptic soy agar (TSA) at 28°C for 48 h and suspended in 0.1M MgSO_4_. Bacterial density (600 nm) was adjusted to 10^9^ CFU mL^−1^. Each plant was inoculated with 8 mL of bacterial suspension, except uninoculated ones that were irrigated with the same quantity of MgSO_4_. After 1 week the plants were inoculated again.

### Plant parameters

The number of flowers and fruits was recorded weekly during all the experiment. Mature fruits were harvested, weighed, measured (large and small diameters), frozen in liquid nitrogen, and stored at −80°C until biochemical/chemical analyses.

For each plant, fresh weights (FW) of root and shoot (without flowers and fruits) were recorded at harvest. Then, each shoot and root system was dried in oven at 60°C for three days and dry weight was (DW) recorded.

Leaf chlorophyll and total carotenoid concentrations were determined spectrophotometrically. Fresh leaf samples (0.03 g from each plant) were kept in the dark at 4°C for 3 days in N, N-dimethyl-formamide (1.5 mL) for pigment extraction. Samples were analyzed according to Porra et al. (1989) using a 0.1–0.5 nm resolution range spectrophotometer.

### Fruit biochemical analyses

In order to investigate the effect of PGPB and AMF combinations on strawberry quality, we randomly chose five plants per treatment (55 plants in total) for the assessment of pH, titratable acids, content of organic acids (malic, quinic, citric, and fumaric), concentration of sugars (sucrose, glucose, and fructose), ascorbic acid (vitamin C), and anthocyanidins (pelargonidin and cyanidin).

pH and total acidity of strawberries were determined diluting the fruit homogenate (5 g from each plant) in deionized, degassed water (30 mL). Samples were centrifuged at 10,000 rpm at room temperature (RT) for 20 min. The supernatant was used for the determination of pH. The same samples were well mixed and 0.1 M NaOH was added dropwise until reaching the titration end-point (pH 8.1) according to official method published by the Italian Government in DM 03.02.1989. Results were expressed as percentage of citric acid per fresh weight unit.

Organic acid concentration was determined by HPLC (Dionex, Sunnyvale, CA, USA; injection volume 20 μL, column Aphera C18 polymer 25 cm × 4.6 mm, 5 μm, column temperature 30°C, flow rate 0.4 mL min^−1^) at 210 nm, according to Keutgen and Pawelzik (2007), with some modifications as reported in Bona et al. (2015).

Ascorbic acid concentration was determined by diluting fruit homogenate (5 g) with 50 mL of deionized water. The solution was shaken at 200 rpm for 10 min at RT, filtered with filter paper and decolorized twice with PolyVinylPyrrolidone (PVP-0.1 g per 10 mL). The pH of the samples ranged between 3.5 and 4.0 in agreement with the instructions of the enzymatic analytical kit (Cat. Nr. 10409677035, R-Biopharm, Roche, Germany). The vitamin C content was measured at 578 nm.

Sucrose, D-glucose and D-fructose concentrations were determined spectrophotometrically at 340 nm using a standard enzymatic analytical kit (Cat. Nr. 10716260035, R-Biopharm, Roche, Germany). Fruit homogenates (5 g) were diluted to 50 mL with deionized water, shaken at 200 rpm for 1 h, filtered through filter paper and decolored twice with PVP. The colorless serum (1 mL) was diluted in deionized water (1/2 v/v) and used for the analyses.

Anthocyanins were extracted from strawberry homogenate (10 g) and separated isocratically by HPLC according to Comandini et al. (2008) with some modifications as reported in Bona et al. (2015). Commercial standards of pelargonidin chloride and cyanidin chloride (Sigma-Aldrich, St. Louis, MO, USA) were used to quantify the content of each anthocyanin according to the equation reported in Chandra et al. (2001); moreover, their identities were also confirmed by MALDI-TOF analysis Voyager DE-PRO (AB Sciex, Framingham, United States).

### Elements extraction and determination

An inductively coupled plasma with mass spectrometry (ICP-MS) X Series II system (Thermo Electron Corporation, Waltham, USA) and an inductively coupled plasma with optical emission (ICP-OES, Spectro Genesis, Kleve, Germany) were used.

Fruit homogenate (1.0 g) was put in an Erlenmeyer flask with nitric acid (4.0 mL, ≥69.0% Trace Select, Fluka) and hydrogen peroxide (2.4 mL, ≥30% Trace Select, Fluka) and subjected to wet digestion for 3 h at 320°C. After cooling, the sample was diluted to 50.0 mL with water and nitric acid before ICP-MS or ICP-OES analysis.

Ca, K, Mg, Na, P, and S were determined by ICP-OES, while Ag, Al, As, Ba, Be, Bi, Cd, Ce, Co, Cr, Cs, Cu, Dy, Er, Eu, Fe, Gd, Hf, Hg, Ho, Ir, La, Li, Lu, Mn, Nd, Ni, Pb, Pd, Pm, Pt, Rb, Ru, Sb, Sc, Se, Sm, Sn, Sr, Ta, Tb, Th, Tl, U, V, Y, Yb, and Zn were determined by ICP-MS.

### Volatile profile

Volatiles were analyzed by head space solid phase microextraction method coupled to gas chromatography-mass spectrometry (HS SPME GC-MS). The GC-MS system (Varian, Walnut Creek, USA) was equipped with HP-INNOWAX capillary column (30 m × 0.25 mm, 0.25 μm, Varian). The extraction was performed using an SPME fiber Divinylbenzene/Carboxen/Polydimethylsiloxane 50/30 μm coating (Supelco, Pennsylvania, USA) after conditioning.

One gram of homogenized sample was added with 1.0 mL sodium chloride 0.10 M, vortexed for 2 min and centrifuged for 10 min at 10,000 rpm at 4°C. The supernatant (1.0 mL) was put in a 20 mL vial sealed by a PTFE/silicone septum; benzaldehyde at 100 mg L^−1^ (Sigma Aldrich, MO, USA) was added as internal standard. Equilibration was performed for 5 min at 33°C, while extraction for 30 min. The fiber was left in the injection part of the GC-MS at 250°C for 20 min. Before sampling, the fiber was reconditioned and blank runs were done periodically to reveal possible carryover effects. Helium was used as carrier gas at 1.0 mL min^−1^; the oven temperature was held isothermal at 35°C for 3 min, raised to 60°C (2°C min^−1^), then from 60 to 130°C (10°C min^−1^), maintained at 130°C for 8 min, raised from 130 to 250°C (20°C min^−1^) and held isothermal for 5 min. MS detection was performed in full scan acquisition from 35 to 350 m/z with electron ionization at 70 eV.

### Statistical analysis, PCA and PCA-DA analysis

Data were analyzed by two-way ANOVA (R software package) using “Fungus” and “Bacterium” as factors. A one-way ANOVA followed by Fisher test (StatView 4.5, Abacus) with significance cut-off at *P* < 0.05 was used to assess differences among the treatments.

Data were also treated by multivariate statistical tools as Principal Component Analysis (PCA) (Massart et al., 1998; Negri et al., 2011) and PCA-Discriminant Analysis (PCA-DA).

In PCA-DA (Hoogerbrugge et al., 1983) the space defined by the Principal Components (PCs) is used to identify linear discriminant functions (LDFs) able to separate the samples in the classes present. In two class problems, only one LDF is provided, able to discriminate the samples in the two classes present.

## Results

### Mycorrhizal colonization

**Figure 1:**
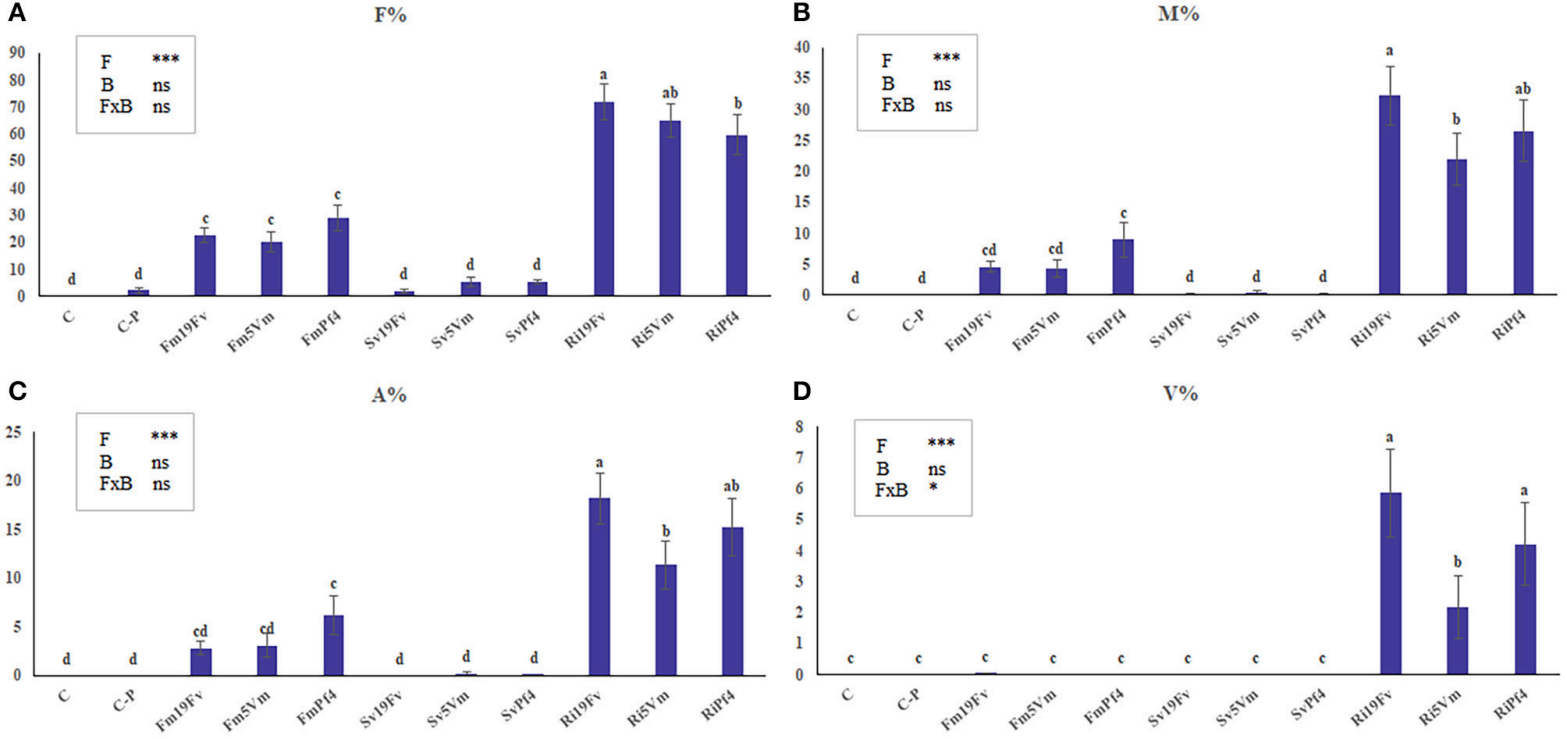
Mycorrhizal colonization in strawberry root. Mycorrhizal parameters of *F*. × *ananassa* roots inoculated with three AMF [*F. mosseae* (Fm), *S. viscosum* (Sv) or *R. irregularis* (Ri)] in combination with three strains of *Pseudomonas* sp. (19Fv1t, 5Vm1k, and Pf4). F%, frequency of colonization **(A)**; M%: intensity of the mycorrhizal colonization **(B)**; arbuscular (A%; **C**) and vesicles (V%; **D**) abundance in the mycorrhizal root. Data (*n* = 10) were analyzed by one-way ANOVA with Fisher test, *p* < *0.05*. Bars represent the standard error and different letters indicate significant differences between the various treatments. Moreover a two-way ANOVA is presented in the inset of each graph, considering the two factors fungus **(F)**, bacterium **(B)** and their interaction (FxB): ns not significant; *p < 0.05; **p < 0.01; ***p < 0.0001.

Only rare traces of mycorrhizal colonization (M% always < 0.1) were detected in C-P uninoculated plants (Figure 1B). The three AMF species differently colonized the strawberry root system (Figures 1A–D). *S. viscosum* poorly colonized strawberry roots, producing extremely rare arbuscules and no vesicles. *F. mosseae* colonization was slightly higher (4<M%<9) and arbuscules, but not vesicles, were observed in all treatments. *R. irregularis* was the best colonizer, showing a significant increase (three-to four-fold) of M% (between 15 and 25%) in respect to Fm-plants; arbuscules and vesicles were always detected. The two-way ANOVA (Figures 1A–D, insets) indicated that co-inoculation with PGPB did not affect AMF colonization, with the exception of vesicle percentage, that was influenced by the fungus-bacterium interaction.

### Effects of AMF and PGPB inoculation on plant growth and production

Plant growth parameters were affected by the combination of low phosphate input and co-inoculation (Table 2).

**Table 2:** Plant parameters.

**Plant**	**Flowers per plant**	**Fruits per plant**	**Total fruit fresh weight per plant (g)**	**Average weight of fruit per plant (g)**	**Fruit large diameter (cm)**	**Fruit small diameter (cm)**	**Root DW (g)**	**Shoot DW (g)**	**Root/Shoot**	**Leaf Chl a (μg ml^−1^)**	**Leaf Chl b (μg ml^−1^)**
C	26.1 ± 2.3bc	15.9 ± 1.6bcd	106.4 ± 6.7bc	7.63 ± 0.41ab	2.970 ± 0.060a	2.420 ± 0.060ab	2.77 ± 0.27bc	14.22 ± 0.71a	0.190 ± 0.010bc	27.90 ± 0.95c	9.80 ± 0.26b
C-P	28.8 ± 2.8bc	16.0 ± 1.5bcd	110.8 ± 6.4abc	8.32 ± 0.46ab	2.980 ± 0.070a	2.430 ± 0.070ab	2.22 ± 0.38cde	13.0 ± 1.304ab	0.160 ± 0.010bcd	30.02 ± 0.86ab	10.63 ± 0.32b
Fm19Fv	27.6 ± 2.7bc	12.8 ± 1.5d	91.8 ± 5.4c	7.50 ± 0.39ab	3.100 ± 0.060ab	2.330 ± 0.060ab	1.61 ± 0.15de	10.34 ± 0.90c	0.160 ± 0.010bcd	32.3 ± 1.3a	11.94 ± 0.34a
Fm5Vm	31.3 ± 2.3bc	18.6 ± 1.0ab	106 ± 11bc	7.51 ± 0.37b	3.000 ± 0.060ab	2.300 ± 0.050b	1.40 ± 0.16*e*	9.82 ± 0.88c	0.150 ± 0.020d	32.20 ± 0.82a	11.63 ± 0.29a
FmPf4	32.1 ± 2.6bc	20.3 ± 1.7a	140 ± 18a	7.69 ± 0.36ab	3.020 ± 0.060ab	2.360 ± 0.050ab	2.05 ± 0.14cde	13.46 ± 0.54ab	0.150 ± 0.010cd	31.2 ± 1.0ab	11.49 ± 0.32a
Sv19Fv	29.2 ± 2.2bc	17.10 ± 0.7abc	120.4 ± 6.7ab	8.62 ± 0.39ab	3.030 ± 0.060ab	2.450 ± 0.050ab	3.71 ± 0.25a	13.39 ± 0.67ab	0.280 ± 0.010a	29.1 ± 1.2bc	10.46 ± 0.39b
Sv5Vm	32.1 ± 2.7bc	16.5 ± 1.8abc	106 ± 10bc	7.24 ± 0.36ab	2.930 ± 0.060a	2.330 ± 0.050ab	3.29 ± 0.53ab	13.50 ± 0.85ab	0.240 ± 0.030a	29.2 ± 1.1bc	10.53 ± 0.50b
SvPf4	39.5 ± 2.5a	20.4 ± 1.7a	126 ± 14ab	7.10 ± 0.30b	2.860 ± 0.050a	2.310 ± 0.040b	2.88 ± 0.31abc	15.40 ± 0.64a	0.180 ± 0.020bc	30.48 ± 0.73ab	10.75 ± 0.30b
Ri19Fv	32.4 ± 1.4b	16.3 ± 1.2bcd	128 ± 12ab	8.74 ± 0.50a	3.280 ± 0.060b	2.560 ± 0.060a	2.25 ± 0.25cd	13.70 ± 0.85ab	0.160 ± 0.010bc	28.9 ± 1.8bc	10.39 ± 0.65b
Ri5Vm	25.9 ± 1.5c	14.40 ± 0.70cd	92.2 ± 5.7c	8.14 ± 0.42ab	3.060 ± 0.060ab	2.420 ± 0.060ab	1.75 ± 0.26de	11.31 ± 0.96bc	0.150 ± 0.010cd	31.2 ± 1.1ab	11.60 ± 0.44a
RiPf4	30.2 ± 1.9bc	19.5 ± 1.4a	127 ± 12ab	7.08 ± 0.32b	3.060 ± 0.060ab	2.320 ± 0.050b	2.69 ± 0.39bc	13.43 ± 0.91ab	0.190 ± 0.020b	28.91 ± 0.78bc	10.38 ± 0.29b
Two way Anova	F^*^ B^*^ F × B ^*^	F ns B^**^ F × B ns	F ns B ^**^ F × B ns	F ns B^**^ F × B ns	F^*^ B ns F × B ns	F ^**^ B ns F × B ns	F^***^ B^*^ F × B ^*^	F^**^ B^**^ F × B ns	F^***^ B ns F × B ^**^	F^*^ B ns F × B ns	F ^**^ B ns F × B ns

Data related to number of flowers and fruits per plant, average of total fruit fresh weight per plant (g), average fruit fresh weight per treatment (g) and fruit large (cm) and small diameters (cm), root and shoot dry weight (g), root/shoot ratio and chlorophyll a and b concentration of strawberry plants inoculated or not with AMF and PGPB (labels related to the different treatments are described in Table 1). Data (means ± standard errors, n = 10) were analyzed by one-way ANOVA with Fisher post-hoc test. Different letters within each column indicate significant differences among the treatments (P < 0.05). The lower row shows data obtained by two-way ANOVA, considering the two factors fungus (F), bacterium (B) and their interaction (F × B); **ns** not significant;

* P < 0.05;

** P < 0.01;

*** *P < 0.0001*.

**Figure 2:**
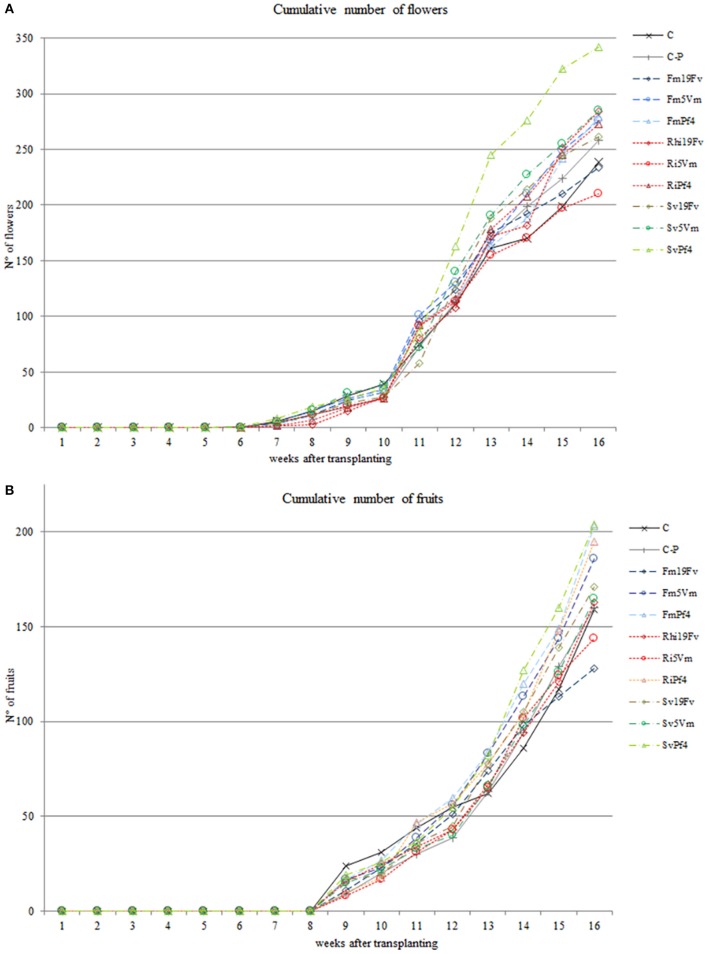
Cumulative number of flowers and fruits. Number of flowers **(A)** and fruits **(B)** produced by strawberry plants inoculated or not with three AMF [*F. mosseae* (Fm), *S. viscosum* (Sv) or *R. irregularis* (Ri)] in combination with three strains of *Pseudomonas* sp. (19Fv1t, 5Vm1k, and Pf4). Flowers were produced for 11 weeks (starting 6 weeks after inoculations), fruits for 8 weeks (starting 9 weeks after inoculations). Data (*n* = 10) were analyzed by one-way ANOVA with Fisher's *post-hoc* test, *p* < 0.05.

The cumulative number of flowers and fruits (Figures 2A,B) was recorded weekly. In SvPf4 plants, inoculation led to a significant induction of flowering (approximately +35%), if compared to the other treatments, especially C-P and Ri5Vm ones, that showed the lowest flower production (Figure 2A). These results were also confirmed by the average number of flowers produced per plant (Table 2). The two-way ANOVA revealed that flowering was affected by all the considered factors (F, B, and F × B). The low input fertilization used in this work (C-P) did not affect flowering and fruit yield compared to control condition (C), while positive effects induced by the inoculated microorganisms were observed (Figures 2A,B). In particular, the strain Pf4 was the most efficient in cooperating with all the used AMF; indeed, an important impact on fruit number (at least +22%) and fruit weight per plant (between +14 and +20%) was observed in all Pf4-plants compared to C-P plants. On the contrary, a significant reduction of total fruit fresh weight was recorded in Ri5Vm and Fm19Fv plants (approximately −17%). Different results were observed for the average fruit fresh weight per plant and for fruit size (large and small diameters) (Table 2): plants inoculated with *P*. *fluorescens* strain 19Fv (Ri19Fv and Sv19Fv) produced on average larger and heavier fruits, but fewer in number (except Ri19Fv) if compared to the Pf4-plants. In agreement with these observations, the two-way ANOVA pointed out the influence of the factor “Bacterium” on fruit size and weight.

In general, inoculation with *S*. *viscosum* increased root DW and root/shoot ratio with the highest values in Sv19Fv plants, that showed significant differences either with plants inoculated with the two other fungi (Fm and Ri) or with uninoculated ones (C and C-P). Similar results were observed for shoot DW, but in this case, SvPf4 plants showed the highest biomass with no significant differences to the controls. The lowest values of root and shoot DW and root/shoot ratio were found in Fm19Fv and Fm5Vm plants, followed by Ri5Vm ones. The two-way ANOVA (Table 2) confirmed that biomass production was affected by both mycorrhizal (F factor) and bacterial (B factor) inoculation. Moreover, concerning root DW and root/shoot ratio, significant differences between the various treatments were attributable to co-inoculation (F × B).

Chlorophyll a and b (Table 2), as well as carotenoid (Table S1) concentrations in leaves were determined. Only the factor fungus (F) strongly improved chlorophyll production. Fm-plants showed the highest values regardless of the used bacterium, with significant differences to Sv- and control (C and C-P) plants, especially for chlorophyll b content. The lowest values of photosynthetic pigment content were observed in C plants. No significant differences between the various treatments were recorded for carotenoid concentration (Table S1).

### Effects of AMF and PGPB inoculation on fruit quality

The pH measured in strawberry fruits ranged between 3.34 (Sv5Vm) and 3.54 (Ri19Fv) (Table 3). Inoculation with *S*. *viscosum* induced a decrease of pH values if compared to the other treatments. The same trend was observed for titratable acidity (expressed as the percentage of citric acid per fresh weight unit—Table 3). The two-way ANOVA suggested that these two parameters were significantly affected both by mycorrhizal (F) and even more by bacterial (B) inoculation (Table 3), but not by the F × B interaction.

**Table 3:** Fruit quality and P concentration.

**Plant**	**pH**	**Titratable acidity**	**Malic acid (mg g^−1^)**	**Sucrose (g Kg^−1^)**	**Glucose (g Kg^−1^)**	**Fructose (g Kg^−1^)**	**Total sugar concentration (g Kg^−1^)**	**P concentration in fruit (g Kg^−1^)**	**P concentration in shoot (g Kg^−1^)**	**P concentration in root (g Kg^−1^)**
C	3.460 ± 0.020abc	0.4510 ± 0.0040abc	0.780 ± 0.090abc	9.0 ± 1.1abcd	16.19 ± 0.87ns	18.01 ± 0.49*a*	43.2 ± 1.8ns	2.86 ± 0.46ns	2.97 ± 0.11c	4.74 ± 0.39bc
C-P	3.500 ± 0.060ab	0.4560 ± 0.0080ab	0.60 ± 0.10bcd	6.4 ± 1.0bcde	11.9 ± 2.6ns	15.1 ± 1.2ab	33.4 ± 3.6ns	3.13 ± 0.31ns	3.29 ± 0.35abc	4.63 ± 0.37bc
Fm19Fv	3.480 ± 0.040ab	0.4530 ± 0.0050ab	0.63 ± 0.12abcd	6.4 ± 1.3bcde	15.78 ± 0.98ns	17.68 ± 0.49*a*	39.9 ± 1.2ns	3.42 ± 0.29ns	3.82 ± 0.23abc	4.68 ± 0.23bc
Fm5Vm	3.480 ± 0.040ab	0.4530 ± 0.0050ab	0.580 ± 0.070bcd	7.1 ± 1.7bcde	11.3 ± 2.2ns	13.6 ± 1.6b	32.0 ± 5.1ns	3.76 ± 0.26ns	3.82 ± 0.24abc	4.19 ± 0.22c
FmPf4	3.440 ± 0.020abcd	0.4480 ± 0.0040abcd	0.790 ± 0.090abc	12.1 ± 2.3*a*	16.56 ± 0.85ns	17.7 ± 1.0*a*	46.4 ± 3.5ns	3.20 ± 0.27ns	3.31 ± 0.17abc	4.29 ± 0.31bc
Sv19Fv	3.460 ± 0.020abc	0.4510 ± 0.0040abc	0.880 ± 0.080*a*	5.24 ± 0.80*de*	14.2 ± 2.6ns	17.6 ± 1.3*a*	37.1 ± 3.9ns	3.09 ± 0.26ns	2.94 ± 0.15c	4.73 ± 0.64bc
Sv5Vm	3.340 ± 0.020d	0.4350 ± 0.0040d	0.770 ± 0.090abc	7.4 ± 1.5*bce*	14.83 ± 0.53ns	16.65 ± 0.74*a*	38.8 ± 2.5ns	2.79 ± 0.21ns	3.07 ± 0.19bc	4.42 ± 0.36bc
SvPf4	3.360 ± 0.020cd	0.4380 ± 0.0040cd	0.820 ± 0.080ab	5.85 ± 0.98cde	15.3 ± 1.6ns	17.63 ± 0.96*a*	38.8 ± 2.3ns	2.99 ± 0.36ns	2.04 ± 0.80d	4.12 ± 0.37c
Ri19Fv	3.540 ± 0.020*a*	0.4610 ± 0.0040*a*	0.530 ± 0.090cd	4.84 ± 0.98*e*	14.4 ± 1.2ns	16.1 ± 1.1ab	35.4 ± 3.0ns	3.64 ± 0.30ns	3.68 ± 0.14abc	5.25 ± 0.27ab
Ri5Vm	3.420 ± 0.050bcd	0.4450 ± 0.0070bcd	0.500 ± 0.080d	9.3 ± 1.9abc	14.47 ± 0.80ns	15.4 ± 1.0ab	39.1 ± 1.6ns	3.05 ± 0.25ns	3.91 ± 0.13ab	6.02 ± 0.32a
RiPf4	3.400 ± 0.050bcd	0.4430 ± 0.0070bcd	0.790 ± 0.080abc	9.1 ± 1.6ab	15.6 ± 1.1ns	16.96 ± 0.83*a*	42.5 ± 3.0ns	2.59 ± 0.34ns	4.13 ± 0.34*a*	5.23 ± 0.33ab
Two way Anova	F^*^ B^**^ F × B ns	F^*^ B^**^ F × B ns	F^*^ B^*^ F × B ns	F ns B^**^ F × B ns	F ns B ns F × B ns	F ns B^*^ F × B ns	F ns B ns F × B ns	F ns B ns F × B ns	F ^***^ B ns F × B ns	F ^**^ B F × B ns

Data related to pH, titratable acidity (calculated as percentage of citric acid on fruit fresh weight), malic acid and sugars (sucrose, glucose, fructose and total sugars) in fruits of Fragaria × ananassa inoculated or not with AMF and PGPB (labels related to the different treatments are described in Table 1). The values of phosphorus concentration in the fruit, shoot and root are also reported. Data (means ± standard errors, n = 10) were analyzed by one-way ANOVA with Fisher post-hoc test. Different letters within each column indicate significant differences among the treatments (P < 0.05). The lower row shows data obtained by two-way ANOVA, considering the two factors fungus (F), bacterium (B) and their interaction (F × B): ns not significant;

* P < 0.05;

** P < 0.01;

*** *P < 0.0001*.

Since organic acids are major components involved in fruit taste, their concentrations were monitored. No differences were detected for quinic, citric, fumaric and ascorbic (vitamin C) acid (Table S1), while the concentration of malic acid significantly changed in the various treatments (Table 3), with the highest values in Sv19Fv-plants. Ri5Vm plants presented the lowest malic acid concentration. The two factors F and B, but not F × B, were responsible for these variations (Table 3).

The concentration of glucose, fructose, and sucrose, the main sugars involved in strawberry taste and flavor, was investigated. Plants fed with a reduced nutrient supply (C-P) produced fruits containing less sucrose, glucose, and fructose than controls (C) (Table 3). No significant differences between the treatments were observed both in the glucose and total sugar concentrations (Table 3 and Table S1), while those of sucrose and fructose changed depending on the various fungus-bacterium combinations.

In general, inoculation with 19Fv bacterial strain, irrespective of fungal inoculation, induced a decrease in sucrose content. Fruits of FmPf4 plants contained the highest level of sucrose. Regarding fructose concentration, C plants showed the highest values followed by plants inoculated with *F*. *mosseae* and *S*. *viscosum* in the presence of 19Fv or Pf4 bacterial strains. Two-way ANOVA indicated that the only relevant factor was bacterial inoculation (B).

**Figure 3:**
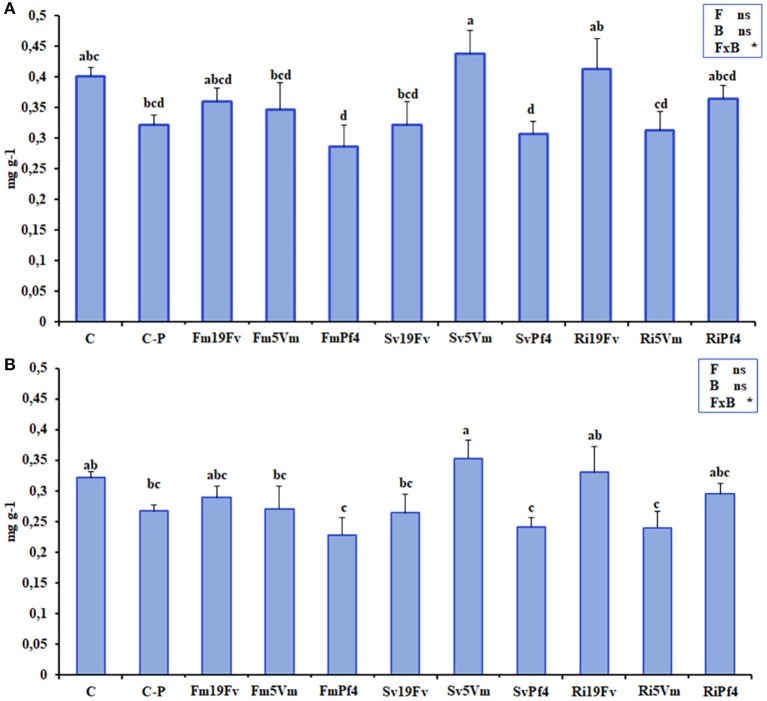
Anthocyanidins. Total anthocyanidin **(A)** and pelargonidin 3-glucoside **(B)** concentrations in fruits of strawberry plants inoculated with three AMF [*F. mosseae* (Fm), *S. viscosum* (Sv) or *R. irregularis* (Ri)] in combination with three strains of *Pseudomonas* sp. (19Fv1t, 5Vm1k, and Pf4). Data (*n* = 5) were analyzed by one-way ANOVA with Fisher test, *p* < *0.05*. Bars represent the standard error and different letters represent significant differences between the various treatments. Table with two-way ANOVA results were reported in the right part of the graphs. The analysis was performed using Fungus (F), Bacteria (B), or the F×B combination as factors: *represent significant results while “ns” not statistically significant differences.

The chromatogram obtained by the HPLC analysis (Figure S1A) of strawberry anthocyanidins showed six peaks, identified, also by MS analysis (Figure S1B), as cyanidin 3-glucoside (peak 1), pelargonidin 3-glucoside (peak 2), pelargonidin 3-rutinoside (peak 3), cyanidin malonyl glucoside (peak 4), pelargonidin malonyl glucoside (peak 5), and pelargonidin acetyl glucoside (peak 6), respectively. The concentration of pelargonidin 3-glucoside significantly varied between the different treatments (Figure 3), displaying the highest values in fruits of Sv5Vm plants that showed marked differences in particular with FmPf4, SvPf4, Ri5Vm, and C-P treatments.

Since pelargonidin 3-glucoside was the most abundant anthocyanidin in strawberry samples, it was also responsible for the total anthocyanidin variation (Figures 3A,B), so the data of these two parameters showed the same pattern and were both affected by the co-inoculation as indicated by two-way ANOVA analysis. The concentrations related to all the other analyzed anthocyanidins did not show any significant differences between the various treatments (Table S1).

### Soil microorganisms influenced element concentration in fruits

**Figure 4:**
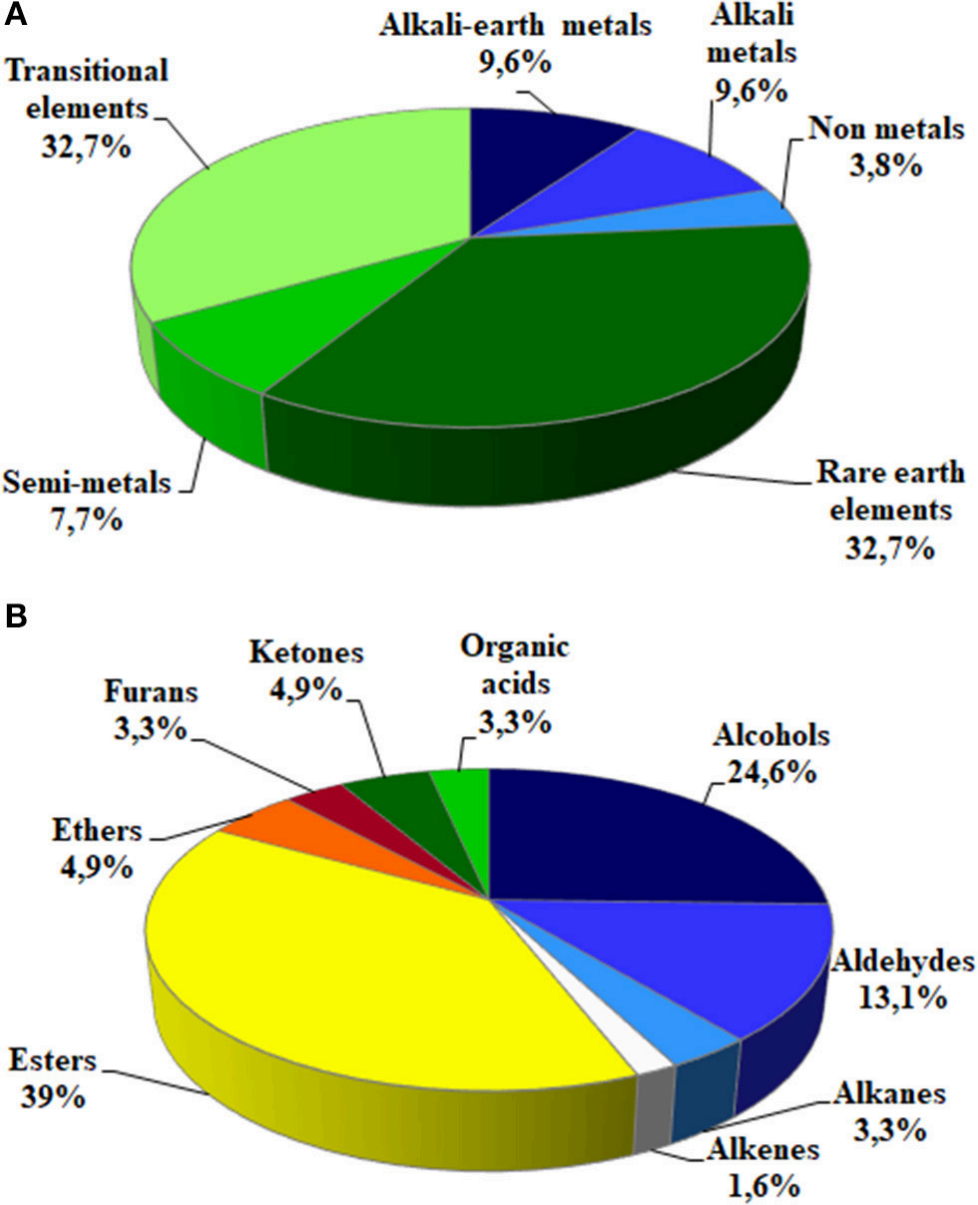
Main representative classes of elements **(A)** and volatiles **(B)** found in strawberry fruits.

A total of 51 elements (mainly belonging to transitional elements, rare earth, alkali and alkali earth metals—Table 4 and Figure 4A) were detected in fruit homogenates. No significant differences due to the inoculation were detected for the concentration of the main nutrients, such as P (Table 3) Na, K, Fe, Mg, Mn, Cu, and Zn (data not shown). On the contrary, P levels significantly changed either in shoots or roots, according also to the mycorrhizal colonization (and therefore according to the fungal species—Table 3 and Table S2): Ri-plants presented an increase of phosphate in both organs, while a decrease was observed in SvPf4 plants that did not show any significant difference with C-P ones.

**Table 4:** List of elements in fruits.

**Element**	**Class**	**Bacterium effect**			**Fungus effect**		
		**5Vm**	**19Fv**	**Pf4**	**Fm**	**Sv**	**Ri**
Li	Alkali metals			√		√	
Be	Alkali-earth metals		√		√		
Al	Transitional elements		√			√	
Sc	Rare earth elements		√			√	
V	Transitional elements		√			√	
Cr	Transitional elements	√			√		
Mn	Transitional elements		√		√		
Fe	Transitional elements		√			√	
Co	Transitional elements	√			√		
Ni	Transitional elements		√		√		
Cu	Transitional elements	√	√		√		
Zn	Transitional elements	√					√
As	Semi-metals			√		√	
Se	Semi-metals		√			√	
Rb	Alkali metals			√		√	
Sr	Alkali- earth metals		√			√	
Y	Rare earth elements	√	√			√	
Rh	Transitional elements	√	√		√		
Pd	Transitional elements	√	√		√		
Ag	Transitional elements	√			√		
Cd	Transitional elements			√	√		
Sb	Semi-metals	√					√
Cs	Alkali metals			√		√	
Ba	Alkali-earth metals	√	√		√		
La	Rare earth elements	√	√			√	
Ce	Rare earth elements	√				√	
Pr	Rare earth elements	√				√	
Nd	Rare earth elements	√	√		√		
Sm	Rare earth elements			√			√
Eu	Rare earth elements		√		√		
Gd	Rare earth elements	√			√	√	
Tb	Rare earth elements	√	√		√		
Dy	Rare earth elements		√		√		
Ho	Rare earth elements	√	√			√	
Er	Rare earth elements	√	√			√	
Tm	Rare earth elements		√			√	
Yb	Rare earth elements	√	√		√	√	
Lu	Rare earth elements		√				√
Ir	Transitional elements	√	√		√		
Pt	Transitional elements	√					√
Hg	Transitional elements		√		√		
Pb	Transitional elements		√				√
Bi	Semi-metals	√	√		√		
Th	Rare earth elements	√	√				√
U	Rare earth elements	√	√				√
Ca	Alkali-earth metals	√			√		
K	Alkali metals	√			√		
Mg	Alkali-earth metals	√			√		
Na	Alkali metals	√	√			√	
P	Non-metals	√			√		
S	Non-metals	√	√			√	

*List of elements positively associated with one of the fungal [R. irregularis (Ri), S. viscosum (Sv) and F. mosseae (Fm)] or/and the bacterial (Pseudomonas sp. 5Vm, P. fluorescens 19Fv and P. fluorescens Pf4) treatments. When there is a positive association, the increasing variable in the considered treatment in comparison with the other treatments is highlighted with a tick (“√”)*.

**Figure 5:**
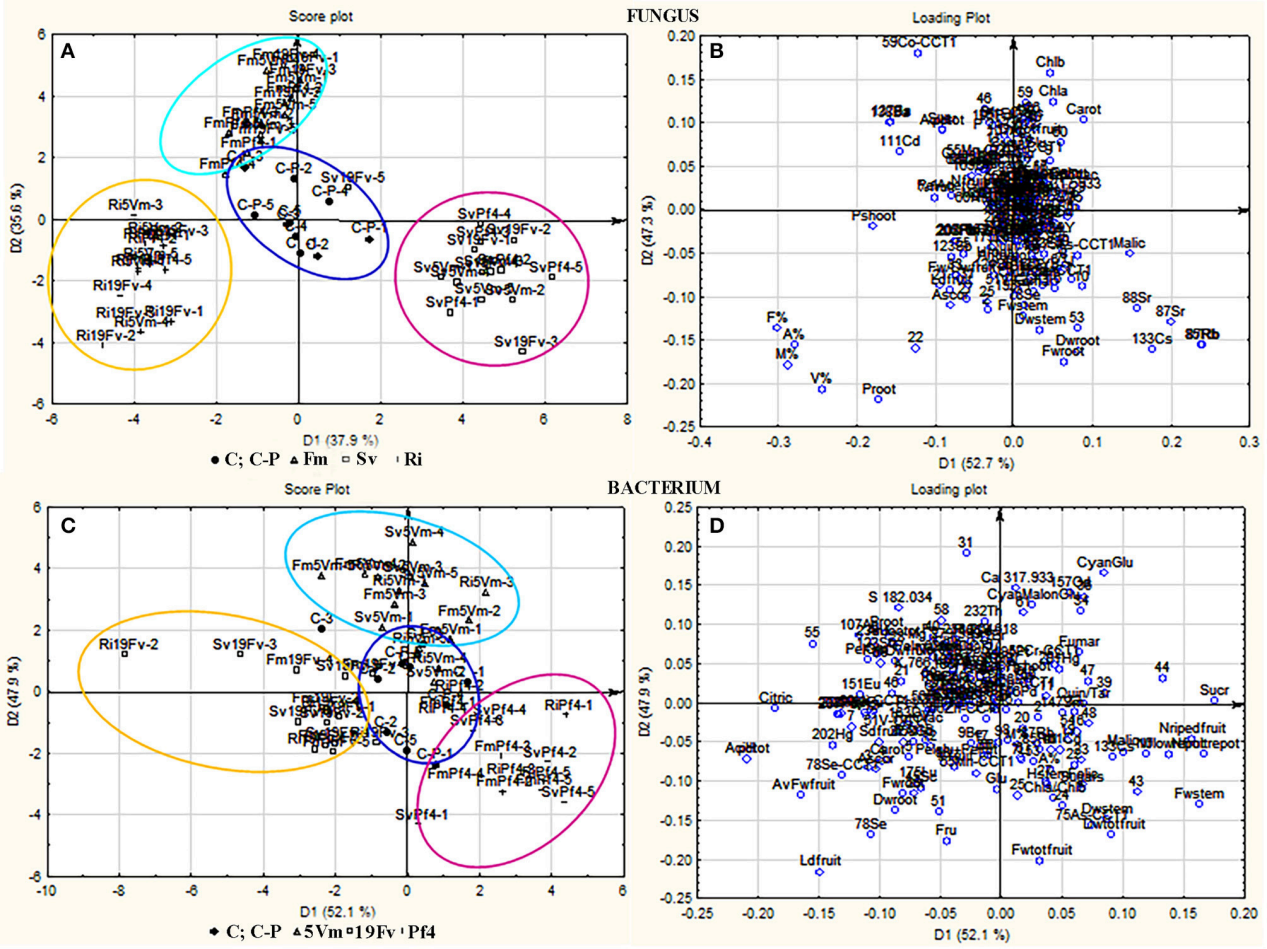
PCA-DA analysis. Score plots **(A,C)** and loading plots **(B,D)** from PCA-DA of volatile compounds, elements and all other parameters recorded in strawberry plants grown or not in presence of different AMF/PGPB mixed inocula. **(A,B)** Samples were separated on the base of the different fungal treatments: *F. mosseae* (F1, marked with Δ and grouped in a light blue circle), *S. viscosum* (F2, marked with □ and grouped in a fuxia circle), *R. irregularis* (F3, marked with + and grouped in a yellow circle); controls (C and C-P) were marked with • and grouped in a blue circle. **(C,D)** Samples were separated on the base of the different bacterial treatments: *Pseudomonas* sp. 5Vm (B1, marked with Δ and grouped in a light blue circle), *P. fluorescens* 19Fv (B2, marked with □ and grouped in a fuxia circle), *P. fluorescens* Pf4 (B3, marked with + and grouped in a yellow circle); controls (C and C-P) were marked with • and grouped in a blue circle.

Concerning the other elements, only Co, Rb, Sr, Cs, and Ba varied significantly in the different treatments (Table 5) and these changes were mainly due to the fungus presence. Co and Ba concentrations were highest in fruits of strawberry plants inoculated with *F. mosseae* independently of the combination with the different bacteria. *S. viscosum* affected the concentration of Sr, Rb, and Cs (Table 5). The concentrations of the last two were also affected by the inoculation with Pf4 (Figure 5, Table 4). In particular, fruits of all Sv-plants showed significantly higher values of Rb, if compared to the other treatments. On the contrary, the lowest Rb concentration was observed in Fm5Vm plants (Table 5). Similar results were obtained from Sr and Cs analyses, although for Cs concentration no significant differences were recorded between Sv and control plants (Table 5).

**Table 5:** Elements and volatiles that significantly varied between the treatments.

	**Co (μg Kg^−1^)**	**Rb (μg Kg^−1^)**	**Sr (μg Kg^−1^)**	**Cs (μg Kg^−1^)**	**Ba (μg Kg^−1^)**	**1,6 heptadien-4-ol #**	**3 methyl-1-butyl acetate #**
C	23 ± 16*c*	(14.5 ± 1.2)10^3^b	(8.3 ± 1.1)10^3^bc	(10.8 ± 1.1)10^1^a	(43.6 ± 6.5)10^2^cd	0.0050 ± 0.0020*c*	0.020 ± 0.011a
C-P	41 ± 25*c*	(14.8 ± 1.3)10^3^b	(8.1 ± 1.2)10^3^bc	97 ± 12ab	(47.2 ± 5.1)10^2^bc	0.0090 ± 0.0010bc	0.0150 ± 0.0070a
Fm19Fv	217 ± 14a	(67.0 ± 7.7)10^2^bc	(65.9 ± 9.7)10^2^c	28.3 ± 8.3*d*	(8.4 ± 1.0)10^3^ab	0.0130 ± 0.0020a	0.0090 ± 0.0040a
Fm5Vm	201 ± 31a	(48.8 ± 8.5)10^2^c	(6.6 ± 1.1)10^3^c	36.5 ± 7.9cd	(9.3 ± 1.4)10^3^a	0.0100 ± 0.0010a	0.0130 ± 0.0030a
FmPf4	207 ± 20a	(73.0 ± 7.8)10^2^bc	(67.8 ± 7.6)10^2^c	50.3 ± 2.6cd	(7.4 ± 1.0)10^3^abc	0.0090 ± 0.0020b	0.0070 ± 0.0010a
Sv19Fv	68 ± 32bc	(35.7 ± 8.8)10^3^a	(13.6 ± 3.2)10^3^a	106 ± 26a	(50.7 ± 7.8)10^2^bcd	0.0100 ± 0.0010a	0.0200 ± 0.0070a
Sv5Vm	– nd	(35.2 ± 2.2)10^3^a	(13.2 ± 1.2)10^3^a	109.4 ± 6.8a	(412.2 ± 5.3)10^1^cd	0.0100 ± 0.0010a	0.0130 ± 0.0050a
SvPf4	– nd	(38.6 ± 4.5)10^3^a	(12.3 ± 2.2)10^3^a	128 ± 12a	(35.2 ± 5.8)10^2^d	0.0090 ± 0.0010bc	0.052 ± 0.017b
Ri19Fv	– nd	(74.3 ± 6.6)10^2^bc	(8.5 ± 2.2)10^2^bc	68 ± 11bc	(8.3 ± 2.4)10^3^cd	0.0070 ± 0.0020bc	0.0110 ± 0.0040a
Ri5Vm	185 ± 58ab	(12.6 ± 3.2)10^3^bc	(7.5 ± 1.2)10^3^c	47.9 ± 4.9cd	(9.0 ± 2.6)10^3^a	0.0100 ± 0.0010a	0.0140 ± 0.0070a
RiPf4	136 ± 34abc	(75.8 ± 6.5)10^2^bc	(72.9 ± 6.4)10^2^c	44.9 ± 7.6cd	(82.9 ± 9.3)10^2^ab	0.0090 ± 0.0010bc	0.0160 ± 0.0050a
Two way Anova	F ^***^ B ns F × B ns	F ^***^ B ns F × B ns	F ^***^ B ns F × B ns	F ^***^ B ns F × B ns	F ^***^ B ns F × B ns	F ^*^ B ns F × B ns	F ^*^ B ns F × B ^*^

Concentration of Co, Rb, Sr, Cs, Ba, 1,6 heptadien-4-ol and 3 methyl-1-butyl acetate in fruits of Fragaria × ananassa inoculated or not with AMF and PGPB (labels related to the different treatments are described in Table 1). The concentrations of the five elements are expressed as μg Kg^−1^ DW, while the concentration of the two volatiles (#) are expressed as the ratio between the peak area of the standard used for the analysis and the peak area of each sample. Data (means ± standard errors, n = 5) were analyzed by one-way ANOVA with Fisher post-hoc test. Different letters within each column indicate significant differences among the treatments (P < 0.05). nd: not detected. The lower row shows data obtained by two-way ANOVA, considering the two factors fungus (F), bacterium (B) and their interaction (F × B): ns not significant;

* P < 0.05;

*** *P < 0.0001*.

### Changes of volatiles in response to the different microbial treatments

Strawberry volatile profile revealed the presence of 58 molecules; of these 55 were identified by comparing their experimental mass spectra with the theoretical ones of compounds present in the library NIST 2.0. They belong to different chemical classes: esters, alcohols, aldehydes, ketones, acids and furanones (Figure 4B): esters (39%), alcohols (24.6%), and aldehydes (13.1%) were the most represented.

When compared individually, only two of these compounds showed a significant variation in relation to microorganism inoculation: 3-methyl-1-butyl acetate (ester) and 1,6-heptadien-4-ol (alcohol) (Table 5). The first one significantly increased in SvPf4 plants compared to all the other treatments (Tables 5, 6). Concerning 1,6-heptadien-4-ol, Fm19Fv plants exhibited the highest concentration with significant differences either to controls (C and C-P) or to all Pf4-plants, regardless of the used fungus.

**Table 6:** List of volatiles in fruits.

**Volatiles**	**PCA-DA number**	**Class**	**Bacterium effect**			**Fungus effect**		
			**5Vm**	**19Fv**	**Pf4**	**Fm**	**Sv**	**Ri**
Methyl acetate	1	Ester		√		√		
Ethyl acetate	2	Ester		√				√
Orthoacetic acid trimethyl ester	3	Ester		√			√	
Dichloromethane	4	Alkane		√		√		
Methyl butanoate	5	Ester		√			√	
Methyl 3-methylbutanoate	6	Ester			√		√	
Benzeneacetaldehyde	7	Aldehyde		√			√	
Butyl acetate	8	Ester		√			√	
Hexanal	9	Aldehyde		√		√		
3-Methyl-1-butyl acetate	10	Ester			√		√	
Butane dien 1,3 acetate	11	Ester			√	√		
Methyl exanoate	12	Ester		√			√	
2-Propylheptanol	13	Alcohol		√			√	
2-Hexenal	14	Aldehyde	√	√		√		
Ethyl hexanoate	15	Ester		√		√		
3-Methylbutyl butanoate	16	Ester			√		√	
Ethyl acetate	17	Ester		√		√		
Octanal	18	Aldehyde		√				√
Cis-3-hexenyl acetate	19	Ester			√		√	
Trans-2-hexenyl acetate	20	Ester	√			√		
2,6-Dimethyl-heptanol	21	Alcohol	√	√				√
1,1-Dimethyloxybenzene	22	Ether			√			√
Cis-2-nonen-1-ol	23	Alcohol			√			√
Tetradecyloxirane	24	Ether			√		√	
2-Butyl octanol	25	Alcohol			√			√
2,4-Hexadienal	26	Aldehyde	√	√		√		
3,7-Dimethyl-3-octanol	27	Alcohol			√			√
Acetic acid	28	Organic acid	√	√			√	
Decyl acetate	29	Ester		√		√		
2-Methylundecanol	30	Alcohol	√	√		√		
Benzaldehyde	std	Aldehyde	√	√				√
Linalool	31	Alcohol			√		√	
Octanol	32	Alcohol		√				√
4-tert-butylcyclohexyl acetate	33	Ester	√			√		
2,4-Dimethyl hexane	34	Alkane		√			√	
4-Methoxy-2,5-dimethyl-3(2H)-furanone	35	Furane	√			√		
Ethyl butanoate	36	Ester	√			√		
Butanoic acid	37	Organic acid	√	√				√
Ethyl pentanoate	38	Ester	√				√	
4-Methylbenzaldehyde	39	Aldehyde	√	√		√		
2-Hexyl-1-decanol	40	Alcohol			√			√
Camphene	41	Alkene			√	√		
Dodecanal	42	Aldehyde			√			√
Phenyl methyl acetate	43	Ester			√	√		
Diocthyl ether	44	Ether	√			√		
5-Ethyl-2-furanone	45	Furane	√	√		√		
Methyl 2-hydroxy-benzoate	46	Ester	√				√	
Isopropyl dodecanoate	47	Ester			√	√		
Dihydropseudoionone	48	Ketone	√	√		√		
1,6-Heptadien-4-ol	49	Alcohol	√				√	
Geranyl isovalerate	50	Ester		√		√		
4-Ethylbenzoic acid, cyclohexylester	51	Ester	√			√		
1-(3,5-Ditert-butyl-4-hydroxyphenyl) ethanone	52	ketone			√		√	
Tridecanol	53	Alcohol	√			√		
Phenol	54	Alcohol	√	√				√
Nerolidol	55	Alcohol		√		√		
Iraldeine	56	Ketone	√	√		√		
2,4-bis (1,1dimethylethyl) phenol	57	Alcohol	√	√		√		
2,6-di-tert-butylhydroquinone	58	Alcohol	√					√

*Volatile compounds positively associated with one of the fungal [R. irregularis (Ri), S. viscosum (Sv) and F. mosseae (Fm)] or/and the bacterial [Pseudomonas sp. 5Vm (5Vm), P. fluorescens 19Fv (19Fv) and P. fluorescens Pf4 (Pf4)] treatments are highlighted with a tick (“√”)*.

### PCA-DA of all analyzed parameters

All the considered variables were analyzed by PCA-DA: the samples were separated on the basis of either “fungal” or “bacterial” treatments (score plot analysis Figures 5A,C, respectively). Each fungal inoculation resulted in well separated clusters from the other fungal treatments, and also from the controls (C, C-P), that occupied the central part of the graph. Strawberries treated with *F. mosseae, S. viscosum*, and *R. irregularis* displayed variable values of different compounds. As far as the fungal inoculants are concerned, the first canonical variable (D1, 37.9% of explained variance) separated plants inoculated with *S. viscosum* from those inoculated with *R. irregularis* or *F. mosseae*, while the second variable (D2, 35.6% of explained variance) separated plants inoculated with *F. mosseae* from those inoculated with *S. viscosum* or *R. irregularis* (Figure 5A).

The pattern of bacterial treatments resulted as well in a generally good separation (score plot Figure 5C), but with a slight overlapping. However, the cluster of plants inoculated with Pf4 was separated from that of plants inoculated with 5Vm by the first canonical variable (D1, 52.1% of explained variance), while the second variable (D2, 47.9% of explained variance) clearly separated plants inoculated with Pf4 from those inoculated with 19Fv.

In addition, loading plots (Figures 5B,D) showed the distribution of all the analyzed parameters in relation to the axes, allowing to correlate the variables with the specific fungal or bacterial treatments. In particular, the colonization parameters (A%, F% M%, and V%) and P concentration in shoot and root were strongly associated to the presence of *R. irregularis* (Figure 5B and Table 7), in accordance with the data presented above. Moreover, *R. irregularis* also modulated some volatiles (ethyl acetate—ester; 1,1-dimethyloxobenzene—eter; 2-butyl octanol, 2,6-dimethyl-heptanol, 3,7-dimethyl-3-octanol, linalool and octanol—alcohols) and chemical elements (Sb—semi metal and Pb—transitional element) (Figure 5B and Tables 4, 6).

**Table 7:** List of different plant and fruit parameters.

**Parameter**	**Bacterium effect**			**Fungus effect**		
	**5Vm**	**19Fv**	**Pf4**	**Fm**	**Sv**	**Ri**
Mycorrhization frequency (F%)	√					√
Mycorrhizal percentage (M%)		√				√
Arbuscule abundance (A%)		√				√
Vesicle abundance (V%)	√	√				√
Root Dry Weight		√			√	
Shoot Dry Weight			√		√	
Root/Shoot Dry Weight		√			√	
Number of flowers			√	√		
Number of fruits			√			
Total fruit fresh weight per plant			√	√		
Average weight of fruit per plant		√			√	
Fruit Large diameter		√				√
Fruit Small diameter		√		√		
Leaf Chlophyll a concentration		√		√		
Leaf Chlophyll b concentration	√	√		√		
Chlorophyll a/Chlorophyll b			√		√	
Carotenoids		√		√		
pH		√		√		
Titratable acidity		√		√		
Malic acid concentration			√		√	
Citric acid concentration		√		√		
Fumaric acid concentration	√				√	
Quinic acid concentration	√				√	
Ascorbic acid concentration		√				√
Total organic acid concentration		√		√		
Glucose concentration		√			√	
Fructose concentration		√			√	
Sucrose concentration	√			√		
Total sugar concentration			√	√		
Cyanidin 3-glucoside concentration	√			√		
Pelargonidin 3-glucoside concentration	√	√				√
Pelargonidin 3-rutinoside concentration		√		√		
Cyanidin malonyl glucoside concentration	√			√		
Pelargonidin malonyl glucoside concentration	√	√				√
Pelargonidin acetil glucoside concentration		√		√		
Total Antocyanidin concentration	√	√				√
P concentration in fruits	√	√		√		
P concentration in shoot	√					√
P concentration in root	√	√				√

*List of plant and fruit parameters positively associated with one of the fungal [R. irregularis (Ri), S. viscosum (Sv) and F. mosseae (Fm)] or/and the bacterial [Pseudomonas sp. 5Vm (5Vm), P. fluorescens 19Fv (19Fv) and P. fluorescens Pf4 (Pf4)] treatments are highlighted with a tick (“√”)*.

*S. viscosum* mainly influenced growth parameters, in particular root and shoot biomass and chla/chlb ratio, besides the content of malic and fumaric acid in fruits. Many volatile molecules changed in response to *S. viscosum* inoculation (Table 6): two aldehydes (benzene-acetaldehyde and 4-methyl benzaldehyde), four esters (methyl 3-methylbutanoate, butyl acetate, 3-methyl-1-butyl acetate and 3-methylbutyl butanoate) and one alcohol (tridecanol). Concerning the elements, Rb, Sr, Cs, Li (alkali metals), Y, Yb (rare earth metals), As, Se (semi metal), and V (transitional element) were the most affected by the inoculation with *S. viscosum*.

Inoculation with *F. mosseae* was strictly related to the increase in photosynthetic pigment concentration (Figure 5B) in accordance with the data presented in Table 2. The fruit acidity (pH, total acidity and citric acid) as well as sweetness (sucrose and total sugar concentration) and some antioxidant compounds (cyanidin glucoside, cyanidin malonyl glucoside) also varied in presence of *F. mosseae*. Six esters (butane dien 1, 3 acetate, trans-2-hexenyl acetate, ethyl butanoate, methyl 2-hydroxy-benzoate and 4-ethylbenzoic acid), two aldehydes (2-hexanal and 2, 4-hexadienal) and two alcohols (2-hexyl-1-decanol, hepta-1,6-dien-4-ol) were associated to *F. mosseae* inoculation, as well as different elements (Table 4) (Co, Cd, Cu, Pd, Ag—transitional elements; Ba—alkali metal; Eu—rare earth metal; P—non metal).

The PCA-DA loading plots also showed that fruit production was mostly influenced by PGPB inoculation (Figure 5D). In particular Pf4 enhanced flower and fruit number and weight, besides increasing shoot biomass. The concentrations of total sugars and malic acid were also positively correlated with Pf4 inoculation, as well as the 3-methyl-1-butyl acetate (ester), phenyl methyl acetate (ester) and tetradecyloxirane (eter) concentration (Tables 6, 7). Concerning the element composition, Pf4 modulated both Cs (alkali metal) and As (semi metal) concentrations in fruits (Table 4).

Inoculation with *P. fluorescens* 19Fv affected fruit size, acidity and sweetness (especially fructose concentration). The aroma and flavor of 19Fv-fruits were mainly due to the following volatiles compounds: orthoacetic acid trimethylester (ester), methyl butanoate (ester), decyl acetate (ester); dichloromethane (alkane); benzeneacetaldehyde (aldehyde), 2-propylheptanol (alcohol), acetic acid (organic acid). The Se (semi metal), Ni (transitional element), Sc, and Eu (rare earth metals) concentrations increased following 19Fv inoculation.

Inoculation with 5Vm influenced chlorophyll b concentration in leaves, besides the content of fumaric acid and the two types of cyanidin (3-glucoside and malonyl glucoside) in fruits (Table 7). The volatile profile was also affected by the inoculation with this bacterial strain that increased the amounts of 2,4-dimethyl hexane (alkane), 4-methyl benzaldehyde (aldehyde), dioctyl ether (ether); ethyl butanoate (ester), isopropyl dodecanoate (ester); 2,4-bis (1,1-dimethyl) (alcohol), linalool (alcohol), nerolidol (alcohol) and 2,6-di-tert-butylhydroquinone (alcohol). In addition to this, element composition of 5Vm fruits was different from that found in 19Fv- and Pf4-inoculated strawberries. In fact, in this case, inoculation affected the concentration of Gd, Fe, Ag (transitional elements), Ca, Mg (alkali earth metals), Th, Ce, Pr (rare earth metals), Sb (semi metal), S, and P (non-metals).

## Discussion

### Plant growth and production

Inoculation with AMF and PGPB differently affected strawberry plant physiology and biochemistry, also increasing yield and improving fruit quality compared to uninoculated plants grown at the same, low, P level. As a consequence, they were similar (and in some cases even better, depending on the inoculated microorganisms and the considered parameter) to control plants grown at the higher P concentration.

In this study, we used three of the most common fungal species that had been found to be associated with roots of strawberry cultivars in many agricultural soils (Santos-Gonzáles et al., 2011), in combination with three bacterial strains previously tested on strawberry individually or in combination with a mycorrhizal consortium (Lingua et al., 2013; Bona et al., 2015). It is well-known that the outcome of the symbiosis is strictly related to the different fungal-plant combinations: the results obtained inoculating different cultivars with the same fungus (and conversely) can be very different (Gosling et al., 2006 and references therein). This is also true in the case of bacteria (Lingua et al., 2013; Guerrero-Molina et al., 2014; Bona et al., 2015).

In our work, mycorrhizal colonization (M%) in AM plants ranged between 0.3 and 32%, depending on the fungal strain, and was in line with the values presented in other studies (Taylor and Harrier, 2001; Stewart et al., 2005; Bona et al., 2015; Tomè et al., 2015). The best root colonizer was *R*. *irregularis*, that generally increased P concentration in both roots and shoots compared to the C-P plants and even to C ones, which were grown at a higher phosphate level. In agreement with various studies (Bucher, 2007; Smith and Smith, 2011; Mensah et al., 2015), higher mycorrhizal colonization was positively correlated with improved P nutrition.

Concerning bacterial inoculation, we did not observe any enhancement of mycorrhization in relation to the three different Pseudomonads (as confirmed by the two-way ANOVA), underlining that, in the present case, they had not a role as MHB; previous reports show that bacterial inoculation can induce decrease (Gamalero et al., 2010) or increase (Pivato et al., 2009) of mycorrhizal colonization, suggesting that effects on root colonization may depend on the different microbial combinations.

Generally, two-way ANOVA suggested that in this experiment the production of shoot and root biomass was significantly affected either by co-inoculation (F × B) or by the factor “Fungus” alone. Although *S. viscosum* poorly colonized the strawberry root system, Sv-plants gave the best results in terms of root and shoot biomass and root/shoot ratio, possibly depending on the combination with the three different bacterial strains.

*F. mosseae*, presenting intermediate values of mycorrhization (if compared to the other two fungi), negatively affected plant biomass, albeit showing a higher concentration of photosynthetic pigments (mainly chlorophyll b). Only the factor “Fungus” influenced this last parameter. As previously shown in different studies, AM symbiosis can affect the production of photosynthetic pigments (Mena-Violante et al., 2006; Baslam et al., 2013; Mo et al., 2016).

In contrast with Bona et al. (2015), that previously reported earlier flowering and fruiting in plants of *F. ananassa* var. Selva inoculated with a “consortium” of AMF (*R. intraradices, G. aggregatum, G. viscosum, Claroideoglomus etunicatum*, and *C. claroideum*) and PGPB (the same of our experiment), we did not observe any variation of these two parameters. While such differences could be due to the use of a different strawberry cultivar (Selva vs. Elyana F1), this trend greatly changed from the 5th week after inoculation, showing an increased productivity in inoculated plants, especially in terms of number of flowers and fruits, both cumulative and per plant, if compared to the controls. In particular, Pf4 enhanced fruit production, regardless of the used fungus, either in comparison to the other bacterial treatments or to the controls. A positive effect of AMF-Pf4 inoculation on the number of fruits and yield had also been observed in Bona et al. (2015). As shown by the two-way ANOVA, the factor “Bacterium” significantly affected fruit number, size, and weight, besides the strawberry quality. The increased yield can be explained by a higher number of flowers and larger fruit size (Bona et al., 2015). Moreover, it is well-known that the phytohormones auxin and gibberellins are involved in strawberry fruit development and growth, especially in the enlargement of receptacle, the main factor determining fruit size and shape (Roussos et al., 2009; Csukasi et al., 2011). Since our PGPB strains are able to produce auxin (Berta et al., 2014; Bona et al., 2015, 2017) we can expect that they were also able to affect the fruit size and development.

### Fruit nutritional quality

Strawberry fruit size, color, and scent are the main factors that influence the consumer to purchase. All these parameters are strictly related to fruit ripeness, a complex process in which the content of sugars, organic acids, vitamins, and volatile compounds greatly changes, affecting the chemical and sensory characteristics of fruits (e.g., pH, total acidity, sweetness, aroma), besides its health-promoting properties (Crespo et al., 2010; Mahmood et al., 2012).

The values of pH recorded in our strawberries were comparable with those found in other varieties (Mahmood et al., 2012; Bona et al., 2015; Concha-Meyer et al., 2016) and reflected the trend of total acidity. The factor “Fungus,” but even more the factor “Bacterium,” significantly modulated these two parameters. Organic acids, partly responsible for the sourness, are also involved in the pH regulation and in the stabilization of anthocyanins, therefore affecting strawberry color (Kallio et al., 2000). The balance between acidity and sweetness plays a key role in the flavor determination (Kallio et al., 2000). Moreover, the concentration of citric and malic acid, the most abundant organic acids in strawberry, can influence the sugar perception. In fact, sweetness is not due to the sugar concentration alone, but also to the low level of organic acids, especially malic (Mikulic-Petkovsek et al., 2007, 2012). According to this, fruits of Ri5Vm plants showed the lowest concentration of malic acid and a high value of sweetness/acidity ratio (Table 3 and Table S1). In general, we detected a significant variation in total acidity and sugar concentration between the various treatments: with few exceptions, the values of acidity were lower in inoculated plants, if compared to C-P ones, leading to an imbalance of sweetness/acidity ratio toward sweetness. The amount of fructose and glucose in fruits was higher than that of sucrose in agreement with different studies reported in the literature (Castellanos-Morales et al., 2010; Akhatou and Fernandez-Recamales, 2014a; Bona et al., 2015). Although the differences between the treatments were not always significant, we observed a general reduction of fructose concentration in fruit of inoculated plants, respect to those of C plants (un-inoculated and grown at higher P level), and this could be explained by the fact that AMF can act as carbon sink (Douds et al., 2000).

FmPf4 fruits had a higher sucrose concentration (Table 3 and Table S1), compared to C-P ones, suggesting that this combination could modulate the production of sugars during photosynthesis. Several studies showed that sugars (mainly sucrose) can influence anthocyanin biosynthesis in fruits (Carbone et al., 2009; Jaakola, 2013; Li et al., 2015). In our work, anthocyanidin concentration was affected by co-inoculation (F × B, as shown by two-way ANOVA), while no effect was attributable to the factors “Fungus” or “Bacterium” alone. Lingua et al. (2013) found a positive effect of microorganism inoculation on cyanidin-3-glucoside and pelargonidin-3-glucoside concentrations. In particular, the 5Vm *Pseudomonas* strain (the same used in this work) gave the best results if used alone. In agreement with these data, we observed an increased amount of pelargonidin-3-glucoside (and also of total anthocyanins, Figure 3) not in all 5Vm-plants but only in those inoculated with *S. viscosum*, the fungus that showed the lowest mycorrhizal colonization. Several studies highlighted a significant variation in strawberry anthocyanin concentration in relation to species, cultivars, genotype, growth conditions (temperature, light, fertilization, irrigation), and degree of ripeness (Carbone et al., 2009; Jaakola, 2013; Li et al., 2015), besides to the fact that microorganism inoculation in soil strongly affects the production of secondary metabolites, depending on the used inoculum (Castellanos-Morales et al., 2010; Lingua et al., 2013; Bona et al., 2015).

### Fruit volatilome

A modulation of volatile/aromatic compound production in response to bacterial/fungal inoculation was observed in many plants of economic interest (Copetta et al., 2006; Bailly and Weisskopf, 2012; Hart et al., 2015; Bona et al., 2016 and references therein). About these molecules, no data on fruits from inoculated strawberry plants were found in the literature. Certain volatile compounds in fleshy fruits are markers of the physiological status of the fruit (un-ripe, ripe, decay) and together with sugar, organic acid and phenolic content, is responsible for their characteristic flavor (Forney, 2001; Prat et al., 2014; Feng et al., 2015; Klee and Tieman, 2018). The volatile profile found in our strawberry was comparable with those recorded in other cultivars (irrespective of the method used for the analysis) with esters as the dominant class, followed by alcohols, aldehydes, ketones, ethers, and other compounds present in small amounts (Forney, 2001; Kim et al., 2013; Samykanno et al., 2013; Prat et al., 2014). About 360 different volatile molecules have been identified in strawberries, and only some of them significantly contribute to the unique strawberry flavor and aroma (Larsen et al., 1992; Prat et al., 2014). The characteristic odor and flavor have been described by five basic sensory notes: caramel, fruity, green, lactone-like, and buttery (Cotton, 2012), that are due to compounds belonging to the classes of molecules mentioned above; variations in the chemical composition and proportion of the different compounds drive the perception changes between cultivars and fruit ripening stages. In our case, the analyzed fruits contained molecules responsible for the abovementioned notes, with the exception of buttery (due in general to the presence of 2,3-butanedione—Schieberle and Hofmann, 1997). Esters and aldehydes contribute to the fresh, green and fruity notes of the strawberry flavor (Latrasse, 1991). In particular, esters are qualitatively the most important compounds in strawberry, providing sweet-fruity and floral characteristics to the taste and odor (Forney et al., 2000; Forney, 2001). In our fruits, methyl butanoate, ethyl butanoate, butyl acetate, methyl hexanoate, and ethyl hexanoate were the most represented esters, in agreement with the data reported in the literature (Ozcan and Barringer, 2011). Esters derive from alcohols and acids and are synthesized by enzymatic or chemical processes. Enzymatic formation of esters in plants is catalyzed by alcohol acyltransferase (AAT): a high activity of AAT produces esters in a large amount, resulting in fruits with strong aroma (Perez et al., 1996). Ester enzymatic biosynthesis occurs during ripening and is not affected by homogenization or by other fruit treatments (Perez et al., 1992; Ozcan and Barringer, 2011). Such reports are in accordance with our results since the fruits were harvested at maturity and no differences were recorded between fresh and frozen samples (data not shown). Among the esters, only the amount of 3-methyl-1-butyl acetate was affected by inoculation with significant differences between SvPf4-plants and all the other treatments.

On the basis of PCA-DA, it was observed that ester concentrations in fruits were mostly affected by FmPf4 and SvPf4 inoculations, suggesting a role for microbial treatments in the determination of the sweet-fruity note in strawberry taste and odor. However, the two clusters of points did not overlap, indicating that the molecules affected in the two treatments were different.

Aldehydes contribute to the green note of flavor, and 2-hexenal is mostly responsible of the intense green-fresh odor (Ulrich et al., 1997) of strawberries. In general, the presence of aldehydes in fruits seems to decrease gradually as ripeness progresses, but studies on different cultivars reported contradictory results (Azodanlou et al., 2004). Unsaturated aldehydes (such as hexanal and its derivatives) are considered as the products of fatty acid oxidation. In our strawberries, 3-hexenal was not detected, while hexanal and 2-hexenal were present in all treatments and their concentration followed the trend [hexanal] < [2-hexenal], in agreement with analyses carried out on various berries (Azodanlou et al., 2004; Du et al., 2011; Hempfling et al., 2013). Moreover, as shown by PCA-DA, inoculation with Fm modulated the expression of unsaturated aldehydes, responsible for the fresh-green note of strawberries, while Sv in combination with 19Fv or 5Vm bacterial strains affected the content of aromatic aldehydes (such as benzeneacetaldehyde and 4-methylbenzaldehyde), mainly responsible for fruity cherry odor and honey, sweet and floral taste, with spicy nuances (http://www.thegoodscentscompany.com/).

Alcohols are one of the most representative classes of compounds in strawberries and this was true also in the present study. Terpenoid alcohols, especially the monoterpene linalool and sesquiterpene nerolidol, positively influence fruit flavor and aroma, providing sweet, floral, citrus-like notes and rose, apple, green notes, respectively (Aharoni et al., 2004). Nerolidol and linalool are present in cultivated strawberries, while olefinic monoterpenes (α-pinene, α-terpineol, ß-myrcene, etc.) are characteristic of the woody, turpentine-like, and resinous odor of wild strawberries (Aharoni et al., 2004; Bianchi et al., 2014); this was in agreement with our results, since no olefinic monoterpenes were found in our fruits. Although no significant differences were recorded for the concentrations of individual alcohols between the various treatments, except for Fm19Fv plants that showed the highest value of 1,6-heptadien-4-ol, PCA-DA indicated that the inoculation with Ri and 5Vm was closely associated to the presence of alcohols in fruits.

### Fruit mineral composition

Strawberries contain many minerals (Perez et al., 1997) that are involved in plant metabolism, besides having an important role in human diet (mainly K, P, Ca, and Mg). We analyzed the elemental composition of strawberry and we detected a total of 51 elements, all at non-toxic concentrations, among which only Ba, Rb, Sr, Cs (alkali metals), and Co (transitional element) significantly varied between some of the treatments. These variations were due to the fungal inoculation (as shown by two-way ANOVA), suggesting that AMF can influence the uptake of these elements and therefore their availability for the accumulation in fruits (Taylor and Harrier, 2001; Hart et al., 2015; Mensah et al., 2015). To our knowledge, no data were recorded in the literature on the effects of microorganism inoculation on alkali metal accumulation in fleshy fruits. PCA-DA showed that the presence of different microorganisms in soil also modulated the fruit elemental composition in different ways. It is noteworthy that each fungus was associated with variations in the concentrations of different elements, and the same was observed also for the three bacterial strains. This trend is in accordance with the data concerning the volatile molecules.

## Conclusions

Present results indicate that co-inoculation with appropriate PGPB and AMF can enhance many physiological parameters and improve strawberry yield and quality, as shown by the increased sugar and anthocyanidin concentrations, the variations of pH and of the concentrations of malic acid, volatile compounds and elements.

The factor “Fungus” mostly affected the parameters associated with the vegetative portion of the plant, while the factor “Bacterium” was especially relevant for fruit yield and quality. Moreover, as shown by PCA-DA, the volatile profile and elemental composition were unique to each treatment, and this diversity was due to the influence of each microorganism combination on the production of secondary metabolites, in particular those related to volatile components. Therefore, fruits from each microbial treatment were different and this can result in unique organoleptic and qualitative characteristics. In the present study, it was shown for the first time that the fruit concentration of some elements and/or volatiles can be affected by the presence of specific microorganisms in the soil.

In addition, our results allow to prospect the selection of optimal plant-microorganism combinations for in-field inoculation, reducing chemical input, leading to economical and ecological soil sustainability and improving fruit production and quality, also in terms of health promoting properties.

## Author contributions

Experimental planning was carried out by GB, DW, and GL. Experimental procedures were mainly carried out by VT and NA, with the help of EB (sample preparation), FG (GC-MS and ICP-OES analyses), EMZ (GC-MS and ICP-OES analyses), FM (HPLC analysis), NM (plant harvest, sample preparation). Statistical data analysis was performed by GL, VT, and ER (multivariate statistical analysis and model interpretation). Data interpretation and overview was carried out by VT, NA, GL, GB, DW, LB, ER, and EMR (the last two for multivariate statistical analysis and model interpretation). The manuscript was written by VT and NL and revised by all the authors.

### Conflict of interest statement

The authors declare that the research was conducted in the absence of any commercial or financial relationships that could be construed as a potential conflict of interest.
