# Supplementary material for: Impact of Beneficial Microorganisms on Strawberry Growth, Fruit Production, Nutritional Quality, and Volatilome
Source: Front Plant Sci. 2018 Nov 16;9:1611. doi: 10.3389/fpls.2018.01611 (PMC6250784; doi:10.3389/fpls.2018.01611)
Supplement: Supplementary file 1 [file Table_1.DOCX]

| **plant** | **Leaf carotenoid (µg ml^-1^)** | **Ascorbic acid**  **(mg/100g)** | **Quinic acid**  **(mg/g)** | **Citric acid**  **(mg/g)** | **Fumaric acid**  **(mg/g)** | **Total sugars**  **(g/Kg)** | **Sweetness/acidity**  **ratio** | **Cyanidin-3-glucoside**  **(mg/g)** | **Pelargonidin 3-rutinoside**  **(mg/g)** | **Cyanidin malonyl glucoside**  **(mg/g)** | **Pelargonidin malonyl glucoside**  **(mg/g)** | **Pelargonidin acetyl glucoside**  **(mg/g)** |
| --- | --- | --- | --- | --- | --- | --- | --- | --- | --- | --- | --- | --- |
| **C** | 0.721±0.024  a | 21.108±3.368  a | 0.763±0.081  a | 2.527±0.117  a | 0.001±8.6x10^-5^  a | 43.244±1.825  a | 10.6±0.6  a | 0.010±0.003  a | 0.007±0.0010  a | 0.002±0.0003  a | 0.056±0.004  a | 0.004±0.0002  a |
| **C-P** | 0.761±0.020  a | 14.775±2.269  a | 1.148±0.321  a | 2.755±0.193  a | 0.001±1.6x10^-4^  a | 33.444±3.597  a | 7.7±1.1  a | 0.005±0.002  a | 0.005±0.0010  a | 0.002±0.0010  a | 0.040±0.003  a | 0.004±0.0001  a |
| **Fm19Fv** | 0.793±0.031  a | 13.030±1.940  a | 0.717±0.050  a | 2.912±0.047  a | 0.001±8.0x10^-5^  a | 39.876±1.204  a | 9.9±0.4  a | 0.007±0.003  a | 0.006±0.0010  a | 0.001±0.0003  a | 0.047±0.004  a | 0.005±0.0003  a |
| **Fm5Vm** | 0.764±0.015  a | 12.422±1.638  a | 0.776±0.078  a | 2.938±0.084  a | 0.001±1.4x10^-4^  a | 32.044±5.114  a | 7.6±1.3  a | 0.012±0.001  a | 0.004±0.0010  a | 0.003±0.0005  a | 0.043±0.007  a | 0.004±0.0004  a |
| **FmPf4** | 0.775±0.021  a | 14.089±1.479  a | 0.684±0.032  a | 2.785±0.074  a | 0.001±9.3x10^-5^  a | 46.383±3.447  a | 10.9±0.9  a | 0.008±0.001  a | 0.005±0.0010  a | 0.002±0.0002  a | 0.038±0.006  a | 0.004±0.0002  a |
| **Sv19Fv** | 0.748±0.026  a | 16.147±2.928  a | 0.708±0.060  a | 2.797±0.123  a | 0.001±6.8x10^-5^  a | 37.061±3.945  a | 8.5±1.0  a | 0.005±0.003  a | 0.005±0.0010  a | 0.001±0.0004  a | 0.040±0.009  a | 0.004±0.0003  a |
| **Sv5Vm** | 0.735±0.024  a | 15.743±2.060  a | 0.750±0.038  a | 2.789±0.089  a | 0.001±1.2x10^-4^  a | 38.832±2.450  a | 9.0±0.5  a | 0.011±0.002  a | 0.006±0.0010  a | 0.002±0.0010  a | 0.060±0.008  a | 0.004±0.0002  a |
| **SvPf4** | 0.775±0.016  a | 12.680±1.143  a | 0.909±0.027  a | 2.723±0.118  a | 0.001±8.6x10^-5^  a | 38.815±2.274  a | 8.8±0.6  a | 0.005±0.002  a | 0.006±0.0010  a | 0.001±0.0002  a | 0.044±0.004  a | 0.004±0.0004  a |
| **Ri19Fv** | 0.729**±**0.041  a | 18.844**±**4.179  a | 0.837**±**0.051  a | 3.095**±**0.043  a | 0.001±6.6x10^-5^  a | 35.399±2.973  a | 7.9±0.5  a | 0.005±0.002  a | 0.006±0.0004  a | 0.002±0.0010  a | 0.062±0.010  a | 0.005±0.0010  a |
| **Ri5Vm** | 0.761**±**0.026  a | 15.675**±**4.560  a | 0.830**±**0.068  a | 2.490**±**0.438  a | 0.001±4.7x10^-4^  a | 39.146±1.586  a | 11.6±2.5  a | 0.005±0.002  a | 0.003±0.0004  a | 0.001±0.0004  a | 0.043±0.006  a | 0.004±0.0002  a |
| **RiPf4** | 0.712**±**0.018  a | 19.876**±**5.238  a | 0.745**±**0.057  a | 2.685**±**0.144  a | 0.001±7.5x10^-5^  a | 42.510±3.045  a | 10.1±0.8  a | 0.010±0.003  a | 0.006±0.0010  a | 0.002±0.0010  a | 0.042±0.006  a | 0.005±0.0003  a |
|  |  |  |  |  |  |  |  |  |  |  |  |  |
| **Two**  **way**  **Anova** | **F ns**  **B ns**  **FxB ns** | **F ns**  **B ns**  **FxB ns** | **F ns**  **B ns**  **FxB ns** | **F ns**  **B ns**  **FxB ns** | **Fns**  **B ns**  **FxB ns** | **F ns**  **B ns**  **FxB ns** | **F ns**  **B ns**  **FxB ns** | **F ns**  **B ns**  **FxB ns** | **F ns**  **B ns**  **FxB ns** | **F ns**  **B ns**  **FxB ns** | **F ns**  **B ns**  **FxB ns** | **F ns**  **B ns**  **FxB ns** |

**Table S1**: **List of biochemical parameters that not significantly varied among the different treatments.** In this table are reported the biochemical parameters that not significantly varied between the different treatments. Labels related to the different treatments are described in Table 1.

Data (means ± standard errors, n=10) were analyzed by one-way ANOVA with Fisher post-hoc test. Different letters within each column indicate significant differences among the treatments (P < 0.05). The lower row shows data obtained by two-way ANOVA, considering the two factors fungus (F), bacterium (B) and their interaction (FxB): ns not significant; *P<0.05; **P<0.01; ***P<0.0001.
