# Supplementary material for: Impact of Beneficial Microorganisms on Strawberry Growth, Fruit Production, Nutritional Quality, and Volatilome
Source: Front Plant Sci. 2018 Nov 16;9:1611. doi: 10.3389/fpls.2018.01611 (PMC6250784; doi:10.3389/fpls.2018.01611)
Supplement: Supplementary file 2 [file Table_2.DOCX]

| **plant** | **P shoot content (mg)** | **P root content**  **(mg)** | **P fruit content**  **(mg)** | **Total P content per plant**  **(mg)** |
| --- | --- | --- | --- | --- |
| **C** | 45.93±3.23  a | 12.89±2.42  abc | 26.02±3.08  a | 84.84±5.94  a |
| **C-P** | 36.38±7.29  a | 9.94±4.11  bcd | 26.60±2.51  a | 72.93±11.20  a |
| **Fm19Fv** | 40.56±6.74  a | 7.79±0.39  cd | 27.79±3.04  a | 76.14±9.59  a |
| **Fm5Vm** | 32.91±3.83  a | 4.59±0.46  d | 26.62±4.21  a | 64.12±5.21  a |
| **FmPf4** | 46.52±3.13  a | 9.08±0.89  bcd | 45.01±12.47  a | 100.61±10.60  a |
| **Sv19Fv** | 39.51±5.08  a | 17.11±2.76  a | 25.75±1.46  a | 82.37±5.42  a |
| **Sv5Vm** | 42.80±4.18  a | 13.04±2.40  abc | 26.14±2.69  a | 81.98±5.50  a |
| **SvPf4** | 52.50±4.33  a | 11.73±1.28  abc | 29.98±3.59  a | 94.20±6.85  a |
| **Ri19Fv** | 52.77±5.18  a | 13.02**±**1.78  abc | 28.87**±**3.75  a | 94.66**±**6.93  a |
| **Ri5Vm** | 50.36±6.86  a | 13.03**±**2.89  abc | 25.31**±**3.55  a | 88.69**±**10.32  a |
| **RiPf4** | 57.99±7.41  a | 14.66**±**2.96  ab | 24.67**±**0.95  a | 97.31**±**10.31  a |
|  |  |  |  |  |
| **Two**  **way**  **Anova** | **F ns**  **B ns**  **FxB ns** | **F ****  **B ns**  **FxB ns** | **F ns**  **B ns**  **FxB ns** | **F ns**  **B ns**  **FxB ns** |

**Table S2**: **Phosphorus content.** In this table are reported either the P content (mg) in different plant organs (shoot, root and fruit) or the total P content (mg) per plant. Labels related to the different treatments are described in Table 1.

Data (means ± standard errors, n=5) were analyzed by one-way ANOVA with Fisher post-hoc test. Different letters within each column indicate significant differences among the treatments (P < 0.05). The lower row shows data obtained by two-way ANOVA, considering the two factors fungus (F), bacterium (B) and their interaction (FxB): ns not significant; *P<0.05; **P<0.01; ***P<0.0001.
