# Supplementary figures and images for: Impact of Beneficial Microorganisms on Strawberry Growth, Fruit Production, Nutritional Quality, and Volatilome
Source: Front Plant Sci. 2018 Nov 16;9:1611. doi: 10.3389/fpls.2018.01611 (PMC6250784; doi:10.3389/fpls.2018.01611)

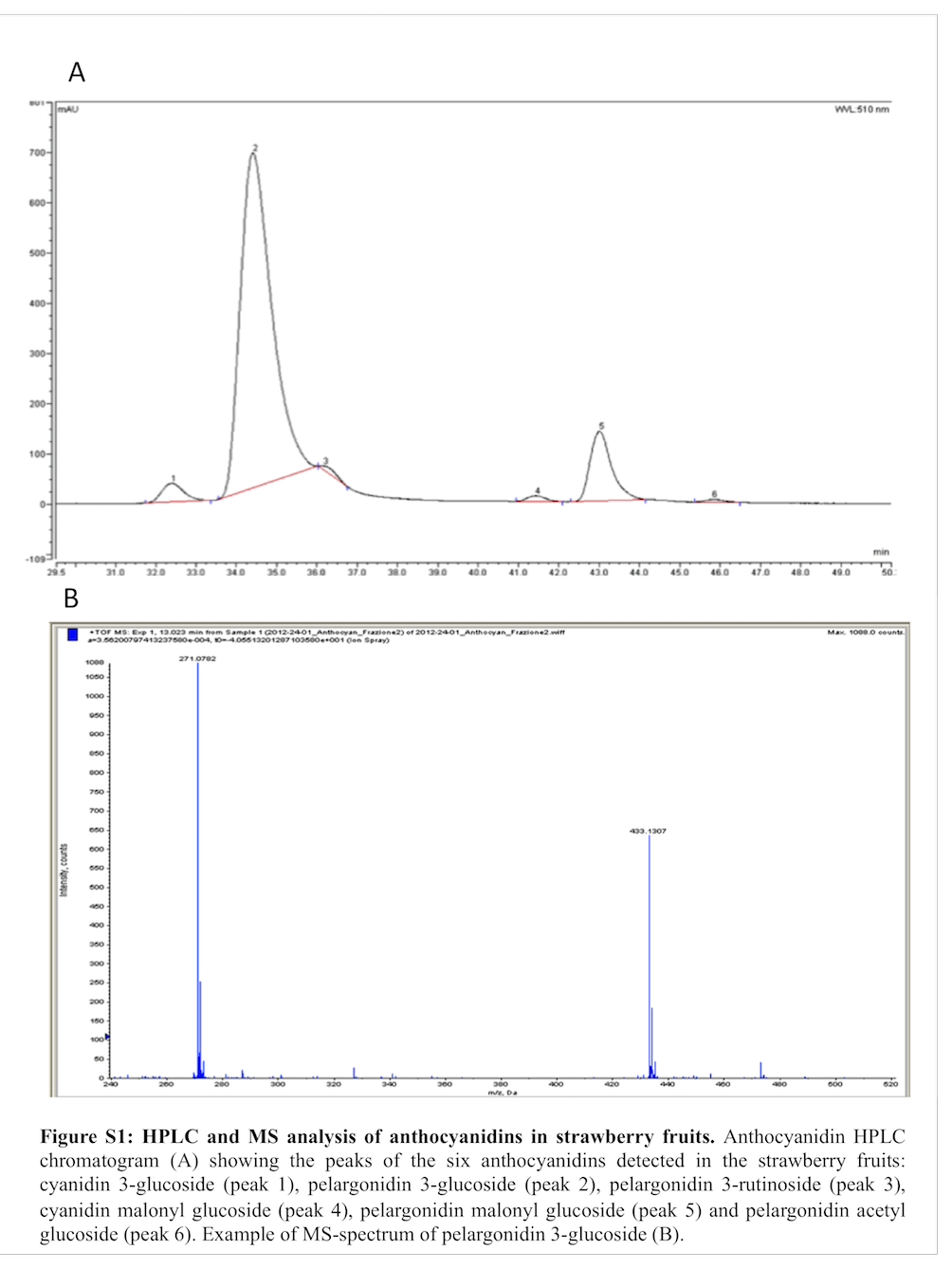

Supplement: Supplementary file 3 [file Image_1.TIFF]
